# Biomarkers for prognosis of meningioma patients: A systematic review and meta-analysis

**DOI:** 10.1371/journal.pone.0303337

**Published:** 2024-05-17

**Authors:** Tin May Aung, Chetta Ngamjarus, Tanakorn Proungvitaya, Charupong Saengboonmee, Siriporn Proungvitaya

**Affiliations:** 1 Centre of Research and Development of Medical Diagnostic Laboratories, Faculty of Associated Medical Sciences, Khon Kaen University, Khon Kaen, Thailand; 2 Department of Epidemiology and Biostatistics, Faculty of Public Health, Khon Kaen University, Khon Kaen, Thailand; 3 Department of Biochemistry, Faculty of Medicine, Khon Kaen University, Khon Kaen, Thailand; 4 Cholangiocarcinoma Research Institute, Khon Kaen University, Khon Kaen, Thailand; National Institute of Cancer Research, TAIWAN

## Abstract

Meningioma is the most common primary brain tumor and many studies have evaluated numerous biomarkers for their prognostic value, often with inconsistent results. Currently, no reliable biomarkers are available to predict the survival, recurrence, and progression of meningioma patients in clinical practice. This study aims to evaluate the prognostic value of immunohistochemistry-based (IHC) biomarkers of meningioma patients. A systematic literature search was conducted up to November 2023 on PubMed, CENTRAL, CINAHL Plus, and Scopus databases. Two authors independently reviewed the identified relevant studies, extracted data, and assessed the risk of bias of the studies included. Meta-analyses were performed with the hazard ratio (HR) and 95% confidence interval (CI) of overall survival (OS), recurrence-free survival (RFS), and progression-free survival (PFS). The risk of bias in the included studies was evaluated using the Quality in Prognosis Studies (QUIPS) tool. A total of 100 studies with 16,745 patients were included in this review. As the promising markers to predict OS of meningioma patients, Ki-67/MIB-1 (HR = 1.03, 95%CI 1.02 to 1.05) was identified to associate with poor prognosis of the patients. Overexpression of cyclin A (HR = 4.91, 95%CI 1.38 to 17.44), topoisomerase II α (TOP2A) (HR = 4.90, 95%CI 2.96 to 8.12), p53 (HR = 2.40, 95%CI 1.73 to 3.34), vascular endothelial growth factor (VEGF) (HR = 1.61, 95%CI 1.36 to 1.90), and Ki-67 (HR = 1.33, 95%CI 1.21 to 1.46), were identified also as unfavorable prognostic biomarkers for poor RFS of meningioma patients. Conversely, positive progesterone receptor (PR) and p21 staining were associated with longer RFS and are considered biomarkers of favorable prognosis of meningioma patients (HR = 0.60, 95% CI 0.41 to 0.88 and HR = 1.89, 95%CI 1.11 to 3.20). Additionally, high expression of Ki-67 was identified as a prognosis biomarker for poor PFS of meningioma patients (HR = 1.02, 95%CI 1.00 to 1.04). Although only in single studies, KPNA2, CDK6, Cox-2, MCM7 and PCNA are proposed as additional markers with high expression that are related with poor prognosis of meningioma patients. In conclusion, the results of the meta-analysis demonstrated that PR, cyclin A, TOP2A, p21, p53, VEGF and Ki-67 are either positively or negatively associated with survival of meningioma patients and might be useful biomarkers to assess the prognosis.

## Introduction

Meningiomas are the most common primary intracranial tumors of the central nervous system (CNS) and arise from the meninges, the protective membrane, that covers the brain and spinal cord [1]. Based on the morphological characteristics, meningiomas are divided into three grades (grade I to III) by World Health Organization (WHO) [2]. About 80% of these are benign, WHO grade I tumors, with an estimated 10-year overall survival 81% [3]. The incidence of grade II and III meningiomas are 15–18% and 2–4%, respectively, of all meningiomas. The grade II and III tumors are difficult to treat due to aggressive growth and high risk of recurrence, often within 5 years. In fact, grade III malignant meningiomas have a poor prognosis, with 10-year overall survival 15% [3]. The clinical manifestation depends on the location and the size of the tumor. The common symptoms are headache, focal cranial nerve deficit, seizure, cognitive change, weakness, vertigo/dizziness, ataxia/gait change, pain/sensory change, proptosis, and syncope, but some cases are asymptomatic [4]. Exposure to ionizing radiation is the only environmental risk factor with the reported risk of ranging from 6 to 10 fold increase of the incidence [5]. High body mass index (BMI), sex hormone therapy, and genetic mutation of NF2, TRAF7, TERT, SMARCB1, PIK3CA, POLR2A, KLF4, AKT1, SMO, SMARCE1, and BAP1 are also risk factors of meningioma [4]. Surgical resection is currently the primary treatment and the best curative option for meningiomas. However, even after surgical resection, tumor recurrence and progression may still occur, leading to reduce the overall survival rate [6, 7]. Several radiological, plasmatic, histological, and molecular prognostic markers have been studied to help stratify meningiomas and provide additional information on prognosis and recurrence. However, imaging techniques are not able to detect tumors until they reach a certain size [8]. The histological grade and the extent of surgical resection are regarded as the two most important prognostic factors of meningioma [9].

Biomarkers in the tissues, blood, and other body fluids are signs of normal or abnormal biological process, condition or disease [10] and the identification of protein biomarkers including immunohistochemical markers have been a great interest for cancer diagnosis and prognosis [11]. Many biological and genetic markers have been examined to predict aggressiveness of meningioma [12, 13]. For example, overexpression of apoptotic marker p53 was associated with poor prognosis in meningioma [14, 15]. The increase of mitotic index using phosphohistone H3 (PHH3) staining in meningioma tissues was associated with poor prognosis of meningioma patients [16, 17]. The Ki-67/MIB-1 labelling index was a biomarker of meningioma associated with higher WHO grade and indicated the risk for recurrence of meningioma [18, 19]. Although various biomarkers have been proposed as potential prognostic markers for meningioma, the results are inconsistent, and their usefulness is under a debate. Therefore, it is important to identify biomarkers which are most relevant for prognosis and the best prospects for personalized treatment in patients with meningioma. In this study, we performed a systematic review and meta-analysis to assess the prognostic biomarkers for patients with meningioma.

## Materials and methods

This study was registered at PROSPERO (International Prospective Register of Systematic Reviews, National Institute for Health Research, https://www.crd.york.ac.uk/PROSPERO) with the registration number CRD42023403315 and carried out in accordance with the Preferred Reporting Items for Systematic reviews and Meta-Analyses (PRISMA), 2020 statement guidelines [20], using the checklist given in S1 File.

### Literature search

To identify relevant published articles that studied the levels of potential biomarker expression as a prognostic factor among individuals with meningioma patients, literature searches were conducted in four different electronic databases: PubMed, CENTRAL, CINAHL Plus, and Scopus with no language restrictions. The last search was carried out on 14^th^ November 2023. The full search term strategies are listed in S1 Table and the search for other resources was done by searching the references of related reviews.

### Study selection

Two authors (T.M.A. and S.P.) independently screened the title and the abstract of all identified studies for eligibility and inclusion. If there were any disagreements in the eligibility of the studies, a discussion was conducted in order to achieve an agreement. Studies were included according to the following criteria: (1) patients were diagnosed as having meningioma with histological confirmation (2) studies included immunohistochemistry-based (IHC) biomarkers with prognostic outcomes [overall survival (OS), progression-free survival (PFS), recurrence-free survival (RFS), or disease-free survival (DFS)] and (3) full-length text of the studies is accessible. Studies excluded are non-clinical ones such as in vitro studies or animal models as well as those unrelated to prognostic biomarkers for meningioma. Studies on the genetic or molecular biomarkers (not IHC biomarkers) for meningioma are also excluded. Review articles (narrative, scoping, systematic reviews, meta-analysis), case-report, conference papers and book reviews were all excluded.

### Data extraction

Extraction of the data was carried out independently by two authors (T.M.A. and S.P.). The extracted data were study characteristics such as first author, publication year, country, sample size, age, sex, WHO grading, follow-up period, candidate biomarker, prognostic outcomes, computed univariable or multivariable hazard ratio (HR) and its 95% confidence interval (CI). We calculated a standard error (SE) of HR from CI or p value if the SE did not report. If the HR was not reported for meta-analysis of biomarkers, we contacted the authors.

### Quality assessment of studies

The quality of the studies included were assessed by two authors (T.M.A. and S.P.) using the Quality In Prognosis Studies (QUIPS) tool [21]. There are six evaluation domains in QUIPS that are used to evaluate the validity and bias of the studies on prognostic factors of meningioma: (1) study participation (2) study attrition (3) prognostic factor measurement (4) outcome measurement (5) study confounding, and (6) statistical analysis and reporting. Since all the studies included in our analysis were retrospective studies, we did not evaluate the items related to study attrition in the second domain, which includes (1) description of attempts to collect information on participants who dropped out (2) reasons for loss to follow-up are provided, and (3) there are no important differences between participants who completed the study and those who did not [21]. The risk of bias was graded as high, moderate or low according to prompting items. If all domains have low risk or moderate risk in only 1 domain, we considered as low, if 2–4 domains have moderate risk as moderate, if at least 5 domains are moderate or at least one domain is high risk, we graded as high risk of bias.

### Statistical analysis

All statistical analyses were performed using Review Manager 5.4.1 [22]. The association between the potential biomarker and its prognostic outcomes of OS, RFS, and PFS was reported by respective HR and 95%CI, respectively. Meta-analysis was used the generic inverse-variance method with a random-effects model to combine results of included studies due to various of the characteristics of participants and cut-off of biomarkers. Heterogeneity within each subgroup was assessed using the I^2^ statistic and Cochrane’s Q test. The subgroup analysis based on WHO grade and cut-off value was employed. Publication bias was evaluated using the funnel plot if there are 10 or more included studies in the meta-analysis of primary outcome (OS).

## Results

### Study selection

**Fig 1:**
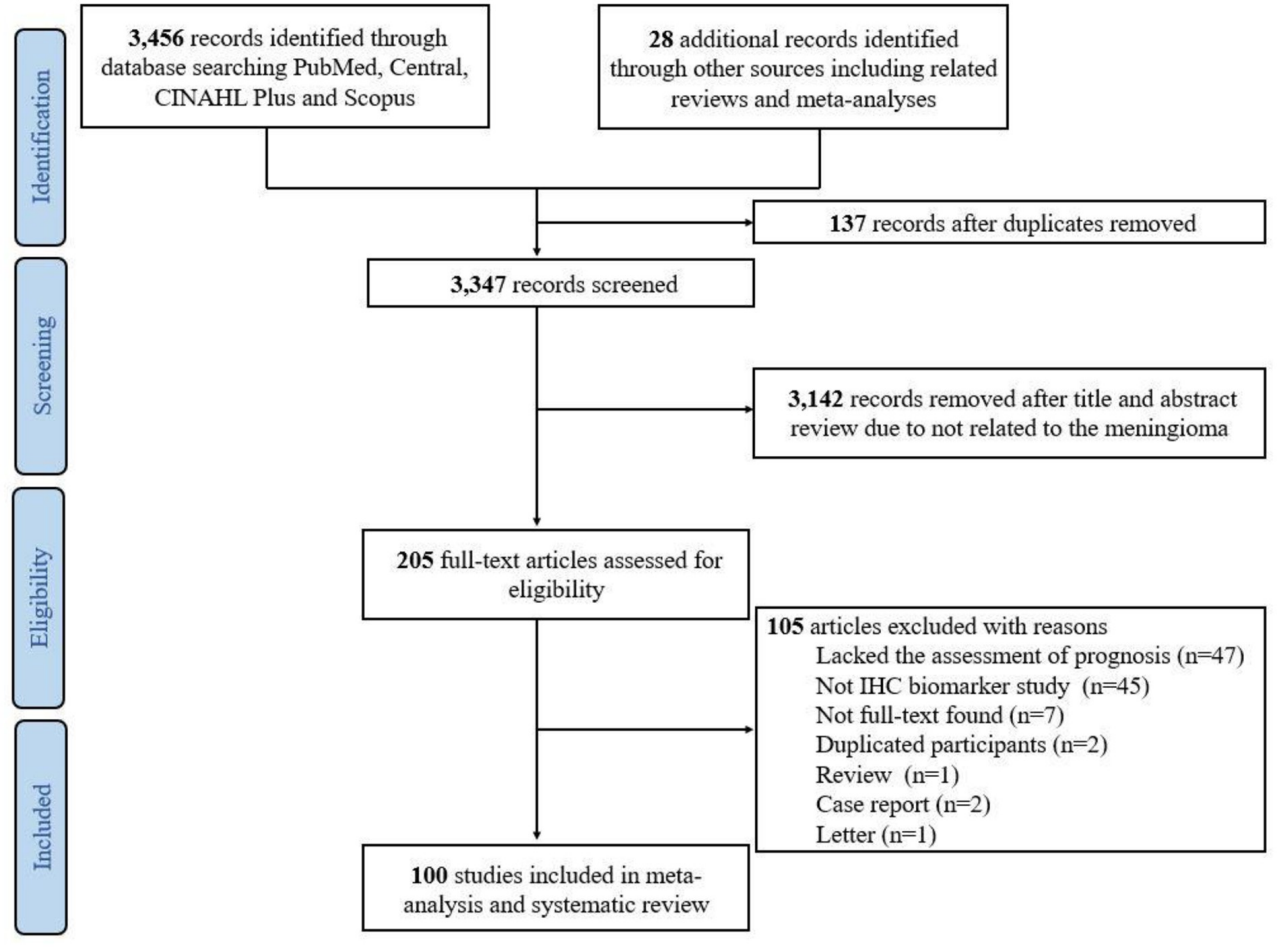
Flow chart of included studies in the systematic review.

A total of 3,484 records were identified after literature search, and 137 duplicates were excluded before screening. After reviewing the titles and the abstracts of the remaining 3,347 records, 3,142 records were excluded. Subsequently, 205 articles were retrieved for full-text screening and their eligibility for systematic review and meta-analysis were assessed. Among these, 104 articles were excluded; 47 articles lacked the assessments of prognosis, 45 articles did not use the IHC biomarker, 7 articles were not available as full-texts, two studies employed duplicate participants, one was a review article, two were case report and one was a letter. Eventually, 100 studies published between 1996 and 2023 were eligible for this systematic review [14–17, 23–118]. Fig 1 illustrates the flow chart of the selection steps of the articles used in this systematic review.

### Study characteristics

The majority of the original articles were from Western countries (61/100, 61%) followed by Asia countries (China, Japan, Korea, Taiwan, India) (39/100, 39%). All the studies in the articles were performed retrospectively. The sample size of the studies ranged from 24 to 1,669 patients and their total was 16,745 patients, with the female predominance. Based on the WHO grading of meningioma, 44 studies included patients with grade I, II, and III, 13 studies included grade I and II, 7 studies included grade II and III, while 11, 12 and 5 studies dealt with only grade I, II, or III respectively. The remaining 8 studies did not specify the grade of meningioma of the patients. To evaluate the prognosis, 11 studies used OS as the endpoint, 46 studies used RFS, 18 studies used PFS, 14 studies used both OS and PFS, 9 studies used both OS and RFS and 2 studies used DFS. The characteristics of the original studies are summarized in S2 Table.

### Quality assessments

**Fig 2:**
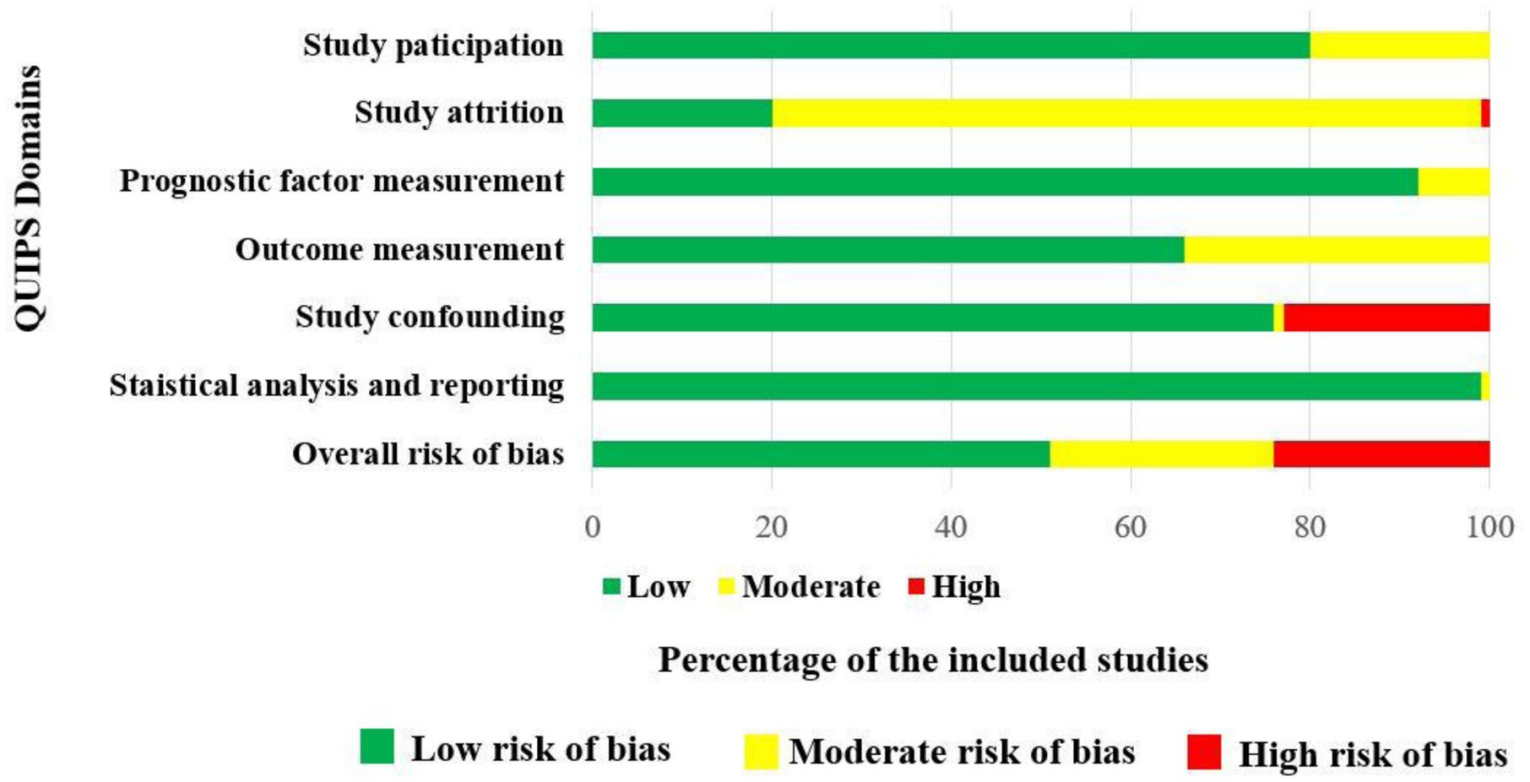
Summary of the quality assessment for the included studies using the six domains of the QUIPS tool for included studies.

QUIPS tool was used to assess the quality of the studies included. Approximately a half (51/100, 51% of the studies included had a low risk of bias. Out of 100 studies, 25 studies (25%) had a moderate risk of bias, mainly due to inadequate description of “Domain 2: study attrition” and “Domain 4: outcome measurements”. The remaining 24 studies (24%) have a high risk of bias, particularly in “Domain 5: study cofounding”. The summary of the quality assessment of the studies included are shown in Fig 2 and the S3 Table.

### Association of biomarkers and prognosis in the meta-analysis

To evaluate the prognostic value of biomarkers of meningioma patients, we performed a meta-analysis of at least two or more studies of the association between 11 biomarkers (PR, cyclin A, TOP2A, p21, MCM6, H3k27me3, Bcl-2, p53, VEGF, PHH3, and Ki-67/MIB-1) and their respective prognostic outcomes using the univariate or multivariate HR and 95% CI by forest plot of random-effects model.

#### Prognostic value of progesterone receptor (PR) expression

**Fig 3:**
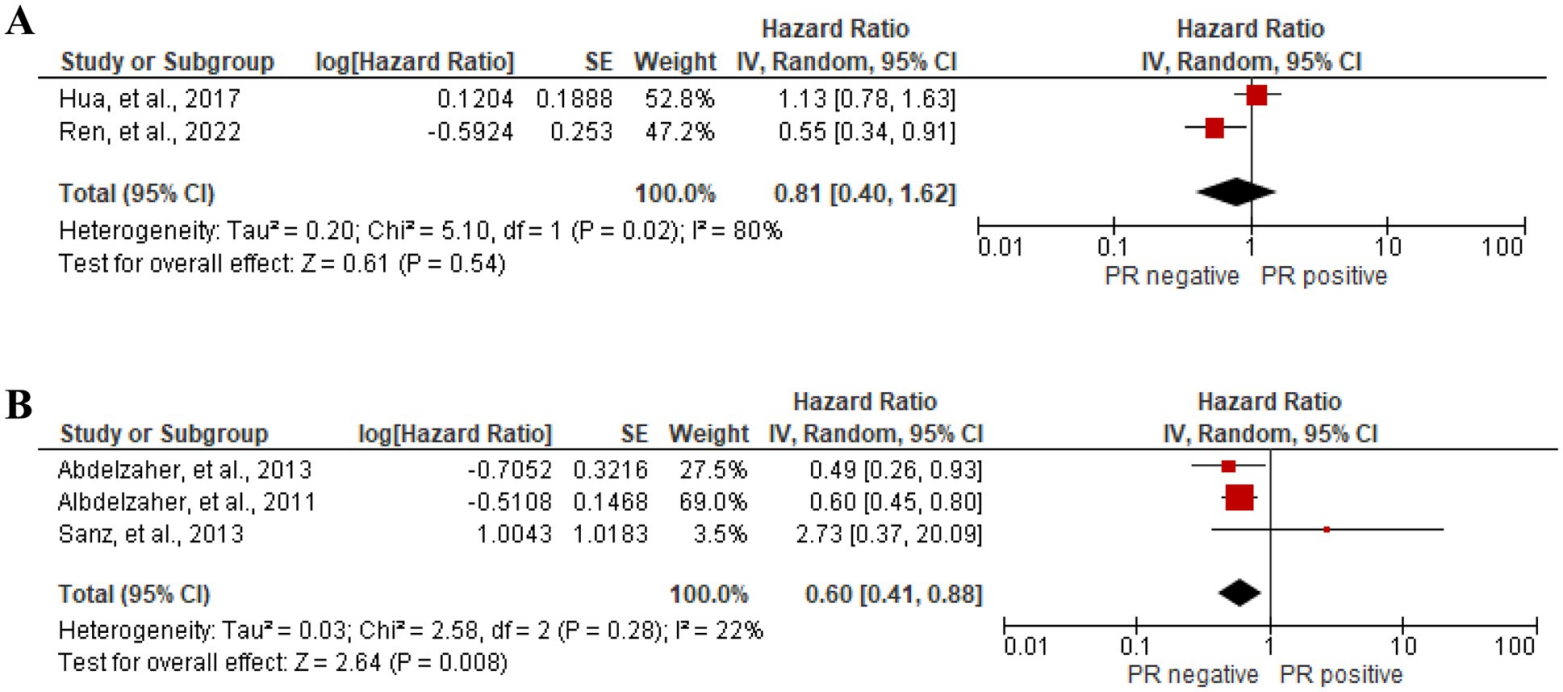
Association between PR expression and (A) PFS, and (B) RFS of meningioma patients by forest plot.

Two studies [54, 96] with a total of 318 participants reported the association between PR expression and PFS of high grade (grade II and grade III) meningioma patients. The forest plot showed no significant association between PR expression and PFS (HR = 0.81, 95%CI 0.40 to 1.62, I^2^ = 80%), Fig 3A. On the other hand, 3 studies [14, 23, 99] with a total of 460 participants indicated the statistically significant association between positive PR expression and good prognosis of RFS of meningioma patients (HR = 0.60, 95%CI 0.41 to 0.88, I^2^ = 22%), Fig 3B. Apart from our meta-analysis, Guadagno et al. [48] reported that high PR expression was associated with good prognosis of OS and RFS of meningioma patients using a Log rank test (p<0.05). However, PR expression was not associated with OS in grade III meningioma patients by univariate cox regression analysis (HR = 0.830, 95%CI 0.486 to 1.418) [54]. PR negative expression was independently associated with poor DFS of meningioma patients (HR = 0.9182, 95%CI 0.854 to 0.9873) [67]. On the other hand, Li et al. [72] reported that PR was not associated with PFS of clear cell meningioma by Log rank test (p = 0.2).

#### Prognostic value of cyclin A expression

**Fig 4:**
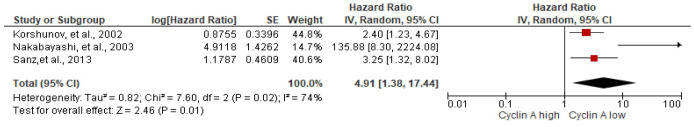
Association between cyclin A expression and RFS of meningioma patients by forest plot.

Fig 4 and S4 Table demonstrate the statistically significant association between high cyclin A expression and poor RFS of 3 studies [68, 81, 99] with 475 meningioma patients in forest plot (HR = 4.91, 95%CI 1.38 to 17.44, I^2^ = 74%).

#### Prognostic value of topoisomerase II α (TOP2A) expression

**Fig 5:**
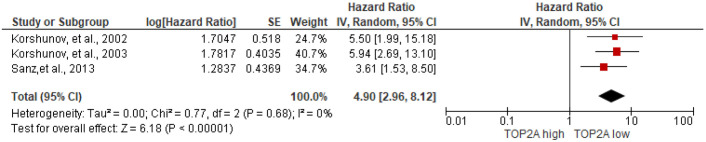
Association between TOP2A expression and RFS of meningioma patients by forest plot.

The potential of TOP2A expression as a biomarker to predict prognosis of 669 meningioma patients in 3 studies [68, 69, 99] was analyzed. The meta-analysis of the forest plot showed a statistically significance of association between high TOP2A expression and poor RFS of meningioma patients (HR = 4.90, 95%CI 2.96 to 8.12, I^2^ = 0%), Fig 5.

#### Prognostic value of p21 expression

**Fig 6:**
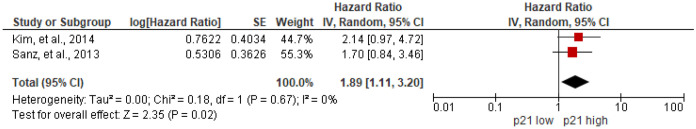
Association between p21 expression and RFS of meningioma patients by forest plot.

Two studies [64, 99] with a total of 202 participants was determined the association between p21 expression and RFS of WHO grade I and II meningioma patients. The meta-analysis showed that low p21 expression was associated with high rate of recurrence in these patients (HR = 1.89, 95%CI 1.11 to 3.20, I^2^ = 0%), Fig 6. Moreover, negative p21 staining was associated with poor RFS of meningioma patients by Log-rank test (p = 0.001) [69].

#### Prognostic value of minichromosome maintenance complex component 6 (MCM6) expression

**Fig 7:**
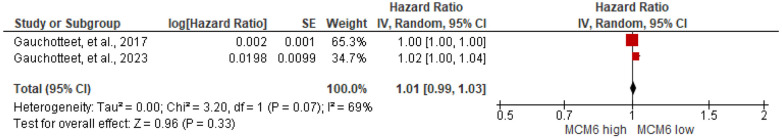
Association between MCM6 expression and PFS of meningioma patients by forest plot.

Meta-analysis of two studies [44, 45] with 154 participants evaluated the association between MCM6 expression and PFS of meningioma patients. The forest plot showed no association between MCM6 expression and PFS of meningioma patients (HR = 1.01, 95%CI 0.99 to 1.03, I^2^ = 69%), Fig 7. Moreover, high expression of MCM6 was associated with shorter OS in grade II meningioma patients by the Log rank test (p = 0.02) [44] and in all grades of meningioma patients by univariate Cox regression analysis (HR = 1.005, 95%CI 1.002 to 1.008) [45].

#### Prognostic value of trimethylation of Histone H3.3 Lysine 27 (H3K27me3) expression

**Fig 8:**
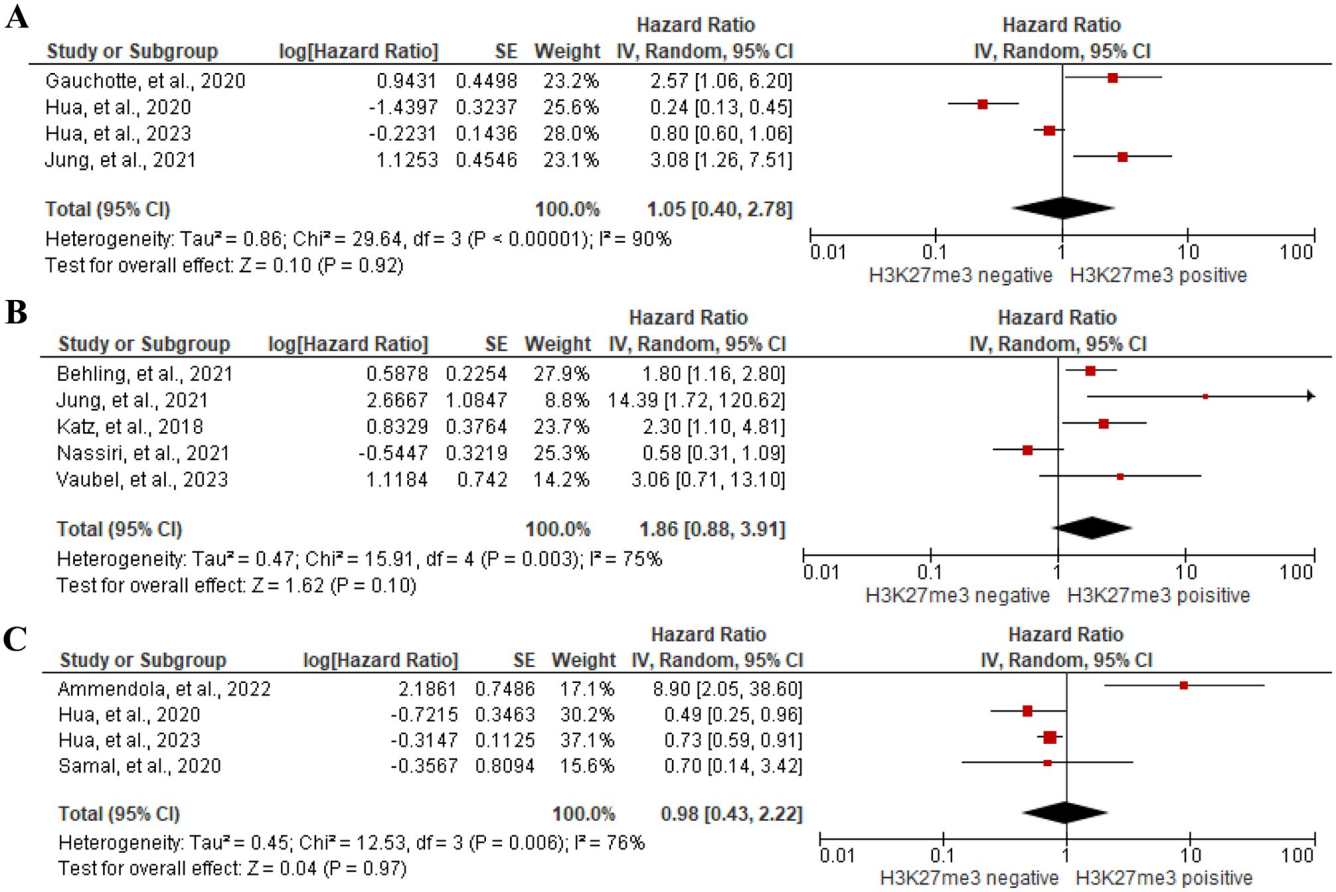
Association between H3K27me3 expression and (A) OS, (B) RFS, and (C) PFS of meningioma patients by forest plot.

We meta-analyzed 4 studies [44, 52, 53, 57] with 563 participants to assess the association of H3K27me3 expression and OS of meningioma patients. For the association between H3K27me3 expression and RFS, 5 studies [30, 57, 60, 85, 106] with 1,855 meningioma patients were meta-analyzed. Moreover, we meta-analyzed 4 studies [24, 53, 98] with 544 participants to determine the association between H3K27me3 expression and PFS. The results of meta-analyses indicated no significant association between H3K27me3 expression and OS (HR = 1.05, 95%CI 0.40 to 2.78, I^2^ = 90%), RFS (HR = 1.86, 95%CI 0.88 to 3.91, I^2^ = 75%), or PFS (HR = 0.98, 95%CI 0.43 to 2.22, I^2^ = 76%) of meningioma patients, Fig 8A–8C. The subgroup analysis showed significant association between H3K27me3 expression and OS (HR = 2.81, 95%CI 1.50 to 5.26, I^2^ = 0%) or RFS (HR = 5.42, 95%CI 1.25 to 23.45, I^2^ = 28%) of high grade meningioma patients, S5 Table. Moreover, Gauchotte et al. [46] showed no significant association between H3K27me3 and PFS of WHO grade III meningioma patients by Log-rank test (p = 0.221). Toland et al. [102] reported the association between high H3K27me3 expression and shorter RFS of meningioma patients using a Log-rank test (p = 0.002).

#### Prognostic value of B cell lymphoma 2 (Bcl-2) expression

**Fig 9:**
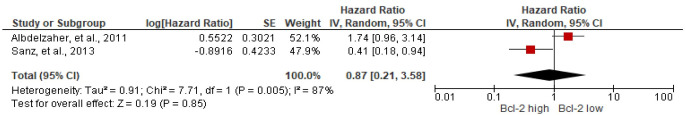
Association between Bcl-2 expression and RFS of meningioma patients by forest plot.

Meta-analysis of two studies [14, 99] with 195 participants showed the association between Bcl-2 expression and RFS of meningioma patients. The forest plot showed no association between Bcl-2 expression and RFS of meningioma patients (HR = 0.87, 95%CI 0.21 to 3.58, I^2^ = 87%), Fig 9.

#### Prognostic value of p53 expression

**Fig 10:**
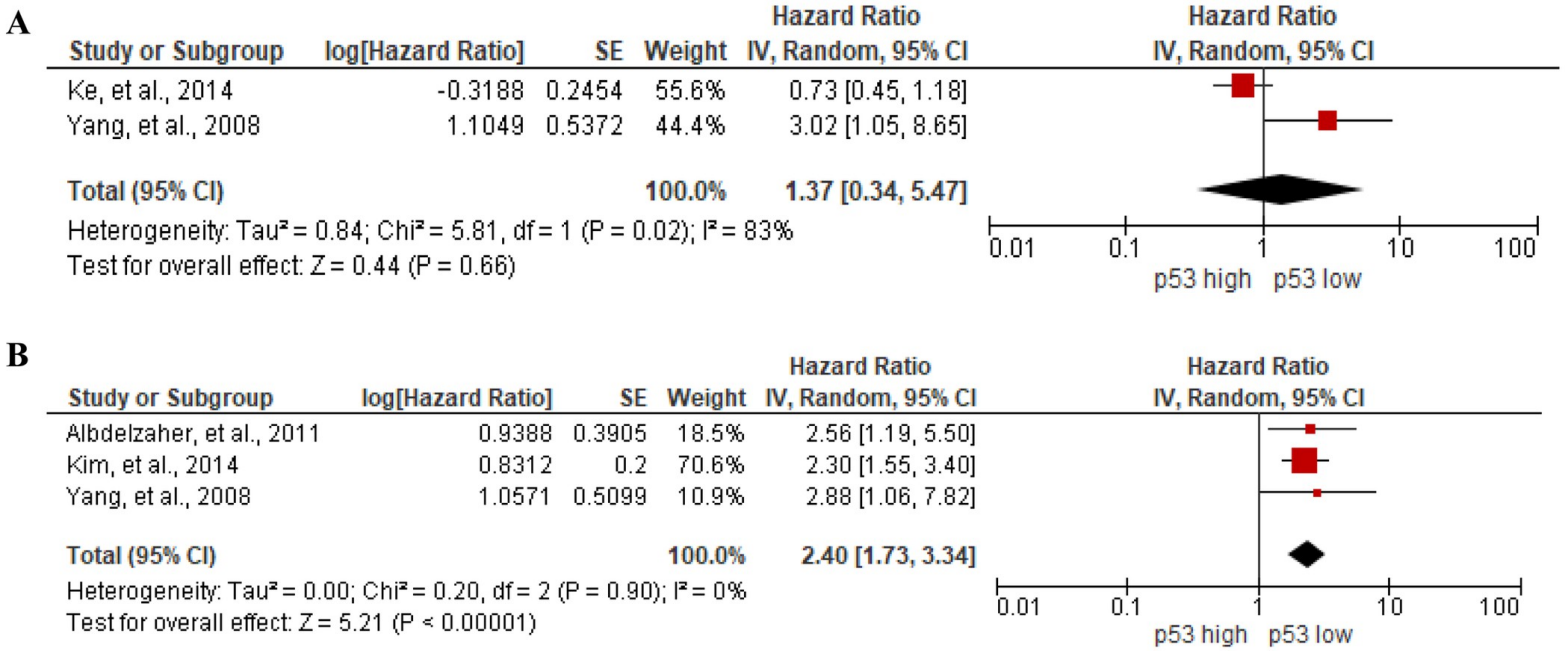
Association between p53 expression and (A) OS, and (B) RFS of meningioma patients by forest plot.

As for the correlation between p53 expression and OS of meningioma patients, meta-analysis of 2 studies [15, 61] with 217 participants showed no significant association between them (HR = 1.37, 95%CI 0.34 to 5.47, I^2^ = 83%), Fig 10A. On the association between p53 expression and RFS of meningioma patients, 3 out of 4 studies [14, 15, 64] with 201 participants showed statistically significant association between high p53 expression and poor RFS (HR = 2.40, 95%CI 1.73 to 3.34, I^2^ = 0%), Fig 10B. However, the remaining one study reported no association between p53 expression and RFS of meningioma patients using a Log rank test (p = 0.0728) [93]. Additionally, Ohba et al. [87] reported significant association between high p53 expression and poor PFS of skull-based meningioma patients by univariate Cox regression analysis (HR = 3.058, 95%CI 1.74 to 5.37).

#### Prognostic value of vascular endothelial growth factor (VEGF) expression

**Fig 11:**
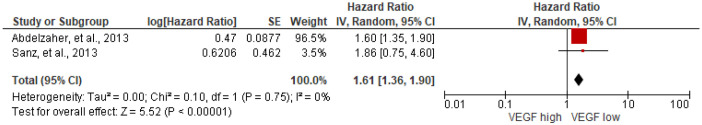
Association between VEGF expression and RFS of meningioma patients by forest plot.

Meta-analysis of 2 studies [23, 99] with 400 participants was determined to evaluate the association between VEGF expression and RFS of meningioma patients. The results showed that high expression of VEGF was significantly associated with poor RFS of meningioma patients (HR = 1.61, 95%CI 1.36 to 1.90, I^2^ = 0%), Fig 11. Likewise, Yamasaki et al. [115] reported the significant association between high VEGF expression and poor RFS of WHO grade I meningioma patients by multivariate Cox regression analysis without HR provided (p<0.0001). In one study, overexpression of VEGF was associated with shorter PFS (HR = 2.22, 95%CI 1.08 to 4.56) but no associated with OS of meningioma patients (HR = 1.3, 95%CI 0.97 to 1.74) [55].

#### Prognostic value of phosphohistone H3 (PHH3) expression

**Fig 12:**
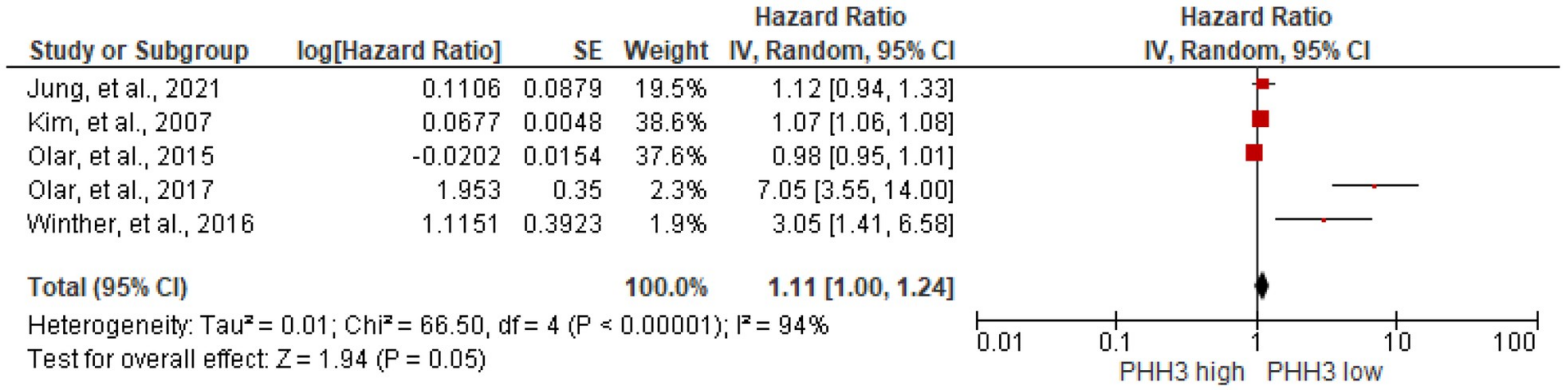
Association between PHH3 expression and RFS of meningioma patients by forest plot.

In the meta-analysis to evaluate the correlation between PHH3 expression and RFS in meningioma patients, five studies [16, 17, 57, 88, 111] with 900 participants are enrolled. The results showed no significant association between them (HR = 1.11, 95%CI 1.00 to 1.24, I^2^ = 94%), Fig 12. Even when correlation between PHH3 staining and the WHO grade and cut-off subgroup showed no association between PHH3 expression and RFS, S6 Table. Similar to our results, Jung et al. [57] revealed that there was no association between PHH3 expression and OS of WHO grade II and III meningioma patients (HR = 1.076, 95%CI 0.952 to 1.217). In the contrary, the positive correlation of PHH3 mitotic index and shorter PFS of WHO grade III meningioma patients was reported (HR = 1.09, 95%CI 1.03 to 1.15) [76].

#### Prognostic value of Ki-67/MIB-1 mitotic index

**Fig 13:**
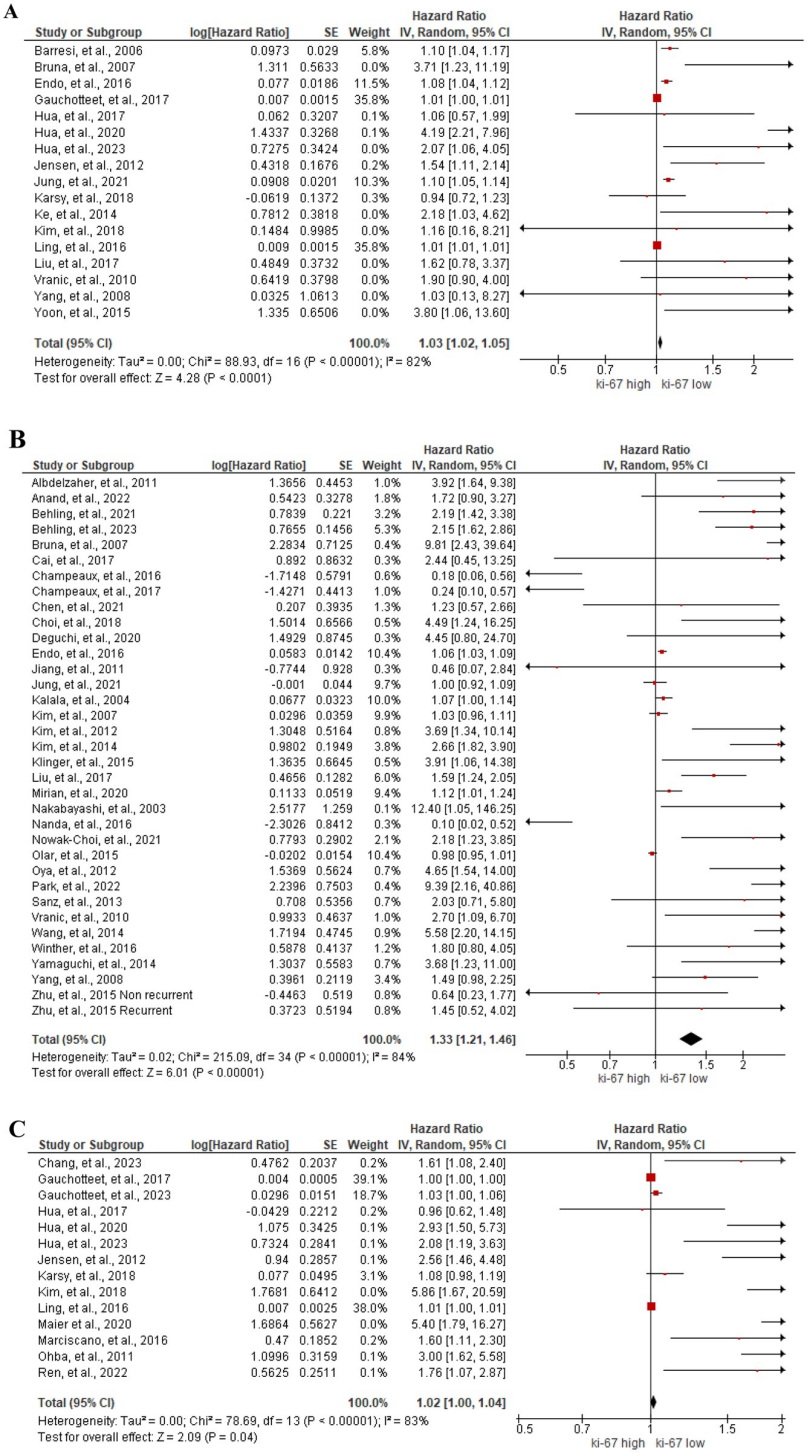
Association between Ki-67 mitotic index and (A) OS, (B) RFS, and (C) PFS of meningioma patients by forest plot.

A total of 23 studies reported the significant correlation between Ki-67 mitotic index and the OS of meningioma patients. Although 7 studies [48, 49, 65, 100, 105, 109, 110] demonstrated the association between overexpression of Ki-67 and poor OS using Log rank test and Cox regression analysis (p<0.05), they did not provide the HR values. Thus, meta-analysis was performed using 16 studies [15, 28, 32, 43, 45, 52–55, 57, 59, 61, 62, 73, 74, 107, 116] with 2,009 patients and the results showed association between high expression of Ki-67 mitotic index and short OS (HR = 1.03, 95%CI 1.02 to 1.05, I^2^ = 82%), Fig 13A. When meningioma patients were subgrouped by WHO grade and cut-off and the association between Ki-67 mitotic index and OS of each subgroup was analyzed, no association was observed either in the low grade or cut-off ≤ 4%. Nevertheless, a significant association with high heterogeneity between Ki-67 expression and OS was found in the subgroup of low and high grade (HR = 1.01, 95%CI 1.0 to 1.03, I^2^ = 84%), high grade (HR = 1.11, 95%CI 1.03, 1.19, I^2^ = 50%), or cut-off > 4% (HR = 1.96, 95%CI 1.29 to 2.96, I^2^ = 80%), as shown in S7 Table. In contrast to our results, Prat-Acin et al. [95] reported that there was no association between Ki-67 expression and OS of WHO grade I plus II meningioma patients by Log-rank test (p>0.05).

Association between Ki-67 mitotic index and RFS of meningioma patients was meta-analyzed using 35 studies [14–17, 25, 30–33, 35, 36, 38, 39, 41, 43, 56–58, 63, 64, 66, 74, 79, 81, 84, 86, 89, 92, 99, 107, 108, 111, 113, 118] with 6,474 participants. The results showed statistically significant association between high Ki-67 mitotic index and poor RFS (HR = 1.33, 95%CI 1.21 to 1.46, I^2^ = 84%), Fig 13B. Subsequently, subgroup analysis of Ki-67 on RFS, showed no heterogeneity in low grade (5 studies, HR = 3.38, 95%CI 2.13 to 5.36, I^2^ = 13%), but substantial heterogeneity was showed in the low and high grade meningiomas (15 studies, HR = 1.24, 95%CI 1.10 to 1.39, I^2^ = 82%) or high grade meningiomas (15 studies, HR = 1.40, 95%CI 1.12 to 1.74, I^2^ = 86%). We also showed the association between Ki-67 and RFS in the subgroup of cut-off ≤ 4% (3 studies, HR = 4.1, 95%CI 2.14 to 7.83, I^2^ = 4%) and > 4% (22 studies, HR = 1.55, 95%CI 1.30 to 1.85, I^2^ = 85%), S7 Table. Although we found 10 studies [48, 49, 65, 68, 78, 82, 93, 97, 100, 101] reporting the association of high Ki-67 mitotic index with poor RFS, (p<0.05), HR was not provided in those studies. Thus, they are not included in the meta-analysis. Meanwhile, 4 studies [40, 67, 100, 115] reported no association between Ki-67 mitotic index and RFS using a Log-rank test (p>0.05).

For the association between Ki-67 mitotic index and PFS of meningioma patients, meta-analysis was performed using 13 studies [37, 44, 45, 52–55, 59, 62, 73, 76, 77, 87, 96] with 2,574 participants. The results showed the association between high Ki-67 expression and PFS of meningioma patients (HR = 1.02, 95%CI 1.00 to 1.04, I^2^ = 83%), Fig 13C. We then analyzed the association between Ki-67 expression and PFS of WHO grade subgroup. The results showed significant association was found only in high grade meningioma, (5 studies, HR = 1.85, 95%CI 1.14 to 3.00, I^2^ = 85%), S7 Table. We also found the significant association between Ki-67 and PFS in the subgroup of cut-off ≤4% and > 4% (4 studies, HR = 1.91, 95%CI 1.1 to 3.29, I^2^ = 85%, and 7 studies, HR = 1.98, 95% CI 1.3 to 3.02, I^2^ = 86%), S7 Table. Although not included in the meta-analysis, 4 studies [29, 49, 51, 83] showed statistically significant association between high Ki-67 expression and poor PFS of meningioma patients without providing the HR (p<0.05). Furthermore, one study revealed the association between high Ki-67 expression and poor DFS of WHO grade I plus grade II patients (HR = 21.7692, p = 0.0032) [27]. Conversely, Li, et al. and Gauchottee, et al. reported no association between Ki-67 mitotic index and PFS of clear cell and grade I meningioma patients by Log-rank test (p>0.05) [44, 72].

#### Publication bias

To determine the publication bias, funnel plot analysis was used for the studies of the prognostic value of Ki-67 for OS. The results showed asymmetry suggesting that the publication bias was found, S1 Fig.

### Association of biomarkers and prognosis in multiples studies

#### Prognostic value of estrogen receptor (ER) expression

Hua, et al. [54] reported the high expression of ER was independently associated with poor prognostic outcomes of OS and PFS of the WHO grade III meningioma patients by multivariate Cox regression analysis (HR = 1.958, 95% CI 1.364–2.809 and HR = 1.515, 95%CI 1.115 to 2.058). However, two studies [14, 67] reported no association between ER expression and RFS of meningioma patients by Cox regression analysis without providing HR value (p>0.05).

#### Prognostic value of cyclin D1 expression

In one study, association of the high expression of cyclin D1 with poor RFS of WHO grade II meningioma patients was reported using univariate Cox regression analysis (HR = 2.02, 95%CI 1.298 to 2.742) [64]. However, another study reported that it was correlated inversely with PFS (HR = 1.001, 95%CI 1.000 to 1.003, p = 0.03) and no correlation with OS of meningioma patients by univariate Cox regression analysis (HR = 1.001, 95%CI 0.999 to 1.003) [45].

#### Prognostic value of human epidermal growth factor receptor 2 (Her 2) expression

Abdelazaher et al. [14] reported that overexpression of Her2 was associated with shorter RFS of WHO grade I meningioma patients using univariate Cox regression analysis (HR = 1.533, 95%CI 1.027 to 2.29). However, two other studies showed that high Her2 expression was not associated with shorter OS or RFS of WHO grade I and II meningioma patients using a Log-rank test (p>0.05) [94, 99].

#### Prognostic value of p16 expression

Low staining of p16 was independently associated with the high rate of recurrence of WHO grade II meningioma patients by multivariate Cox regression analysis (HR = 3.214, 95%CI 2.012 to 4.416) [64]. Korshunov et al. [69] also reported that negative staining of p16 was associated with poor RFS of meningioma patients using a Log-rank test (p = 0.01).

### Association of biomarkers and prognosis in a single study result

Table 1 is a summary of the prognosis outcomes of meningioma patients using biomarkers appeared in a single study alone [26–28, 31, 33, 34, 42, 45, 47–51, 55, 56, 58, 61, 64, 67, 69–71, 75, 80, 90, 91, 98, 99, 103–105, 108, 110, 111, 114]. Among them, high expression of SOX2 was associated with poor OS and PFS of meningioma patients [42, 45]. In the study of Cardona, et al. overexpression of VEGFR2 was associated with shorter OS and PFS. On the other hand, no expression of PDGFRβ was associated with better OS and PFS [34]. Moreover, expression of HuR, HIF-1α, and Glut-1 was associated with OS, but no associated with PFS of meningioma patients [45, 55]. Likewise, high p40 and SP1 expression was associated with poor RFS but no associated with OS of meningioma patients [48, 75]. In addition, 4 biomarkers (Cav-1, PDL^+^CD68^-^, LASS2, MCM7) were associated with OS [28, 50, 61, 112], 10 biomarkers (TXNIP, UbcH10, PCNA, CDK4, CDK6, pRB, p18, Cox-2, TIMP-2, and AKT2) with RFS [33, 56, 58, 64, 69, 99, 108], and 11 biomarkers (KPNA2, CRM1, EGFR ECD Ab, TGF-α, CD20^+^, AKAP12, RB1S780 phosphorylation, EZH2, PR^+^Bcl-2^-^, hENT1, and dCK) with PFS of meningioma patients [47, 49–51, 90, 91, 98, 110, 114]. Furthermore, high expression of p-CREB was associated with poor DFS of meningioma patients [27]. Nevertheless, expression of androgen receptor (AR) was not associated with DFS of meningioma patients [67].

**Table 1:** Biomarkers in meningioma evaluated for association with prognosis outcomes from single studies.

**Study (Reference)**	**Biomarkers**	**Cut-off / Comparison**	**Sample size**	**WHO grade**	**Outcomes**	**Method**	**Statistical analysis**	**HR (95%CI)**	**P value**
Assimakopoulou, et al., 2023 [26]	Polycystin-2	Low/High	82	GI, GII, GIII	OS	IHC	Log-rank	-	>0.05
Barresi, et al., 2015 [27]	p-CREB	score > 40	47	GI, GII	DFS	IHC	Log-rank	-	0.0019
Barresi, et al., 2006 [28]	Cav-1	Score ≥ 6	62	GI, GII	OS	IHC	Log-rank	-	0.039
Behling, et al., 2023 [31]	S100	Positive/Negative	1,669	GI, GII, GIII	RFS	TMA IHC	Cox regression	0.89 (0.56–1.41)	0.614**
Cai, et al., 2017 [33]	TXNIP	<Median/≥Median	65	GI, GII, GIII	RFS	IHC	Cox regression	0.115 (0.02, 0.674)**	0.017**
Cardona, et al., 2019 [34]	VEGFR2	> 10%	31	GII, GIII	OS	IHC	Log-rank	-	0.019
					PFS			-	0.0293
	PDGFRβ	>1%			OS			-	0.0045
					PFS			-	<0.001
Di Bonaventura, et al., 2022 [42]	SOX2	>25%	87	GI, GII, GIII	OS	IHC	Log-rank	-	0.0001
					PFS		Log-rank	-	<0.0001
Gauchotteet, et al., 2017 [45]	HuR	-	85	GI, GII, GIII	OS	IHC	Cox regression	1.001 (1.000, 1.001)*	0.006*
					PFS		Cox regression	1.000 (1.000, 1.000)*	0.17*
Gousiaas, et al., 2014 [47]	KPNA2	<5%/≥5%	108	GI, GII, GIII	PFS	IHC	Cox regression	5.692 (2.64, 12.273)**	<0.001**
	CRM1	<60%/≥60%			PFS		Cox regression	2.584 (1.209, 5.52)**	0.014**
Guadagno, et al., 2016 [48]	p40	>10%	72	GI, GII	RFS	IHC	Log-rank	-	0.0017
					OS		Log-rank	-	0.5529
Guillaudeau, et al., 2012 [49]	EGFR ECD Ab	Low,Interm/Strong	69	GI, GII, GIII	PFS	IHC	Log-rank	-	0.04
	EGFR ICD Ab	No,Low/Interm					Log-rank	-	0.8
Han, et al., 2016 [50]	CD20^+^	-	96	GI, GII, GIII	PFS	TMA IHC	Cox regression	0.966 (0.887, 0.999)**	0.041**
	PDL-1^+^CD68^-^				OS		Cox regression	1.263 (1.067, 1.496)**	0.014**
Hsu, et al., 1998 [51]	TGF-α	≤4/>4	57	GI, GII, GIII	PFS	IHC	Log-rank	-	0.0023
Jensen, et al., 2012 [55]	HIF-1α	-	263	GI, GII, GIII	OS	IHC	Cox regression	3.36**	0.01**
					PFS		Cox regression	1.32*	0.09*
	Glut-1				OS		Cox regression	1.4*	0.02*
					PFS		Cox regression	1.08*	0.73*
	CA-IX				OS		Cox regression	1.1*	0.31*
					PFS		Cox regression	1.31*	0.18*
**Study (Reference)**	**Biomarkers**	**Cut-off / Comparison**	**Sample size**	**WHO grade**	**Outcomes**	**Method**	**Statistical analysis**	**HR (95%CI)**	**P value**
Jiang, et al., 2012 [56]	UbcH10	-	47	GI, GII, GIII	RFS	IHC	Cox regression	2.918 (1.807, 13.928)**	0.044**
Kalala, et al., 2004 [58]	PCNA	-	125	GI, GII, GIII	RFS	IHC	Cox regression	1.18 (1.03, 1.1)**	<0.0001**
Ke, et al., 2014 [62]	LASS2	-	143	GI, GII, GIII	OS	IHC	Cox regression	1.78 (1.028, 3.083)**	0.04**
Kim, et al., 2014 [64]	CDK4	≥30%/<30%	67	GII	RFS	IHC	Cox regression	2.071 (1.429, 2.713)*	0.039*
	CDK6	≥17%/<17%					Cox regression	3.427 (2.437, 4.417)**	<0.001**
	pRB	≥25%/<25%					Cox regression	2.854 (1.839, 3.869)**	0.008**
	p15	<73%/ ≥73%					Cox regression	1.783 (0.934–2.632)**	>0.05**
	P27	<43%/ ≥43%					Cox regression	1.415 (0.859–1.971)**	>0.05**
Konstantinidou, et al., 2003 [67]	AR	Positive/Negative	51	GI, GII, GIII	DFS	IHC	Cox regression	-	>0.05**
Korshunov, et al., 2003 [69]	p18	Positive/Negative	271	GI, GII, GIII	RFS	IHC	Log-rank	-	0.00001
	P14, p27, p73	Positive/Negative					Log-rank	-	>0.05
Koschny, et al., 2015 [70]	RTRAIL-R2, RTRAIL-R4, Caspase-8, cFLIP, Bak, Bcl-XL, Mcl-1	-	37	GI, GII, GIII	OS, PFS	IHC	Kaplan-Meier	-	>0.05
Kuo, et al., 2019 [71]	Beclin	Score ≥ 4	77	GI, GII, GIII	OS	IHC	Log-rank	-	0.087
	LC3B						Log-rank	-	0.256
Liu, et al., 2021 [75]	SP1	Score ≥ 4	74	GI, GII, GIII	RFS	IHC	Log-rank	-	0.025
					OS		Log-rank	-	0.647
Moutafidi, et al., 2021 [71]	HST1, TRPV1, TRPA1	Low/High	71	GI, GII	OS	IHC	Log-rank	-	>0.05
Parada, et al., 2018 [90]	AKAP12	>0.1	75	GI, GII, GIII	PFS	TMA IHC	Kaplan-Meier	-	0.001
Parada, et al., 2020 [91]	RB1 S780phosphorylation	High/Low	140	GI, GII, GIII	PFS	TMA IHC	Cox regression	2.93 (1.41, 6.09)**	0.004**
**Study (Reference)**	**Biomarkers**	**Cut-off / Comparison**	**Sample size**	**WHO grade**	**Outcomes**	**Method**	**Statistical analysis**	**HR (95%CI)**	**P value**
Samal, et al., 2020 [98]	EZH2	Negative/Positive	149	GI, GII	PFS	IHC	Cox regression	5.29 (1.04, 26.84)**	0.04**
	DNMT-1, DNMT-3A, DNMT-SB						Cox regression	-	>0.05*
Sanz, et al., 2013 [99]	Cox-2	Positive/Negative	135	GI, GII	RFS	TMA IHC	Cox regression	4.94 (1.91, 12.73)**	0.001**
	TIMP-2						Cox regression	9.07 (1.16, 70.6)**	0.035**
	pAKT, Cadherin E, Caspase 3a, β-catenin, Cathepsin D, CD44, EGFR, Her2, MDM2, MMP9, PDGF, PTEN, PR, Survivin, β-TGF,						Cox regression	-	>0.05*
Tsai, et al., 2016 [103]	Nrf2	score ≤70/>70	69	GI, GII, GIII	OS	TMA IHC	Log-rank	-	0.09
Tsai, et al., 2018 [104]	DDX3X	score >60/ ≥60	71	GI, GII	OS	TMA IHC	Log-rank	-	0.09
Ulgen, et al., 2019 [105]	EMA^+^PR^+^S100^-^	-	366	GI, GII, GIII	OS, RFS	IHC	Log-rank	-	>0.05
Wang, et al., 2014 [108]	AKT2	Low/High	94	GI, GII, GIII	RFS	IHC	Cox regression	3.644 (1.607, 8.265)**	0.002**
Wang, et al., 2013 [110]	PR^+^Bcl-2^-^	-	30	Papillary meningioma	PFS	IHC	Log-rank	-	0.011
Winther, et al., 2017 [111]	MCM7	<8%/≥8%	160	GI, GII	OS	TMA IHC + IHC	Cox regression	3.27 (1.43, 7.5)**	0.005**
Yamamoto, et al., 2021 [114]	hENT1	Low/High	45	GI, GII, GIII	PFS	IHC	Log-rank	-	0.0188
	dCK						Log-rank	-	<0.0001

GI, WHO grade I; GII, WHO grade II; GIII, WHO grade III; TMA, Tissue microarray; IHC, Immunohistochemistry

* Outcome of univariate Cox regression analysis,

** Outcome of multivariate Cox regression analysis

Moreover, expression levels of Beclin, LC3B, HST1, TRPV1, TRPA1, Nrf2, and DDX3X did not associate with OS of meningioma patients (p>0.05) [71, 80, 103, 104]. Similarly, the expression of CA-IX and Bcl-2 members family (regulators of apoptosis in tumor cells) including RTRAIL-R2, RTRAIL-R4, caspase-8, cFLIP, Bak, Bcl-XL, and Mcl-1 did not associate with OS or PFS of meningioma patients (p>0.05) [55, 70]. Additionally, co-expression of EMA^+^PR^+^S100^-^ did not associate with OS or RFS in total resection meningioma patients [105]. Moreover, there was no association between expression of EGFR ECD Ab, and DNA methyltransferase (DNMT-1, DNMT-3A and DNMT-SB) and PFS of meningioma patients [49, 98]. Furthermore, several biomarkers including polycystin-2, S100, p14, p15, p27, p73, pAKT, cadherin E, caspase 3a, β-catenin, cathepsin, CD44, MDM2, MMP9, PDGF, PTEN, survivin, and β-TGF did not associate with RFS (p>0.05) [26, 31, 64, 69, 99] and considered not suitable as the prognostic biomarkers for meningioma patients.

## Discussion

Meningioma is the most frequent primary intracranial tumors, accounting for approximately 39% of all brain tumors [119]. Surgical resection is the first option to cure meningioma, but those developed at surgically inaccessible locations, incompletely resected and/or having aggressive histological features (WHO grade II and III) tend to grow progressively or recurrence [120]. In this review, we conducted the systematic review and meta-analysis on the prognostic value of IHC biomarkers for meningioma patients. We identified 11 biomarkers (PR, cyclin A, TOP2A, p21, MCM6, H3k27me3, Bcl-2, p53, VEGF, PHH3 and Ki-67/MIB-1) correlated with prognostic outcomes of OS, RFS, and PFS of meningioma patients by meta-analysis of at least two or more studies. Although, CDK6, KPNA2, and Cox-2 gave promising results (HR >3.0, p<0.001), they were investigated in single studies.

The prevalence of meningiomas is higher in females, with a sex ratio estimated at 2:1 (female:male). This epidemiological data suggests evidence supporting the hormonal influence on meningioma development [121]. Sex hormones such as estrogen and progesterone in both men and women influence etiopathogenesis and growth of various cancers including endometrium, breast, prostate, and lung [122]. One study showed that grade I meningioma patients was associated with high PR expression while grade II with low PR expression [123]. Our systematic review and meta-analysis revealed that higher PR expression was associated with good RFS of high grade meningioma patients (grade II and III). This finding was consistent with a recent systematic review in which high PR expression was associated with favorable prognosis of meningioma patients (grade I, II and III) [124]. Additionally, our meta-analyses showed that PR did not associate with PFS of WHO grade III meningioma patients. Since, the role of PR in tumorigenesis of meningioma remains unknown, needed further study is required for better understanding of the role of PR in prognosis of meningioma patients [125].

Cyclin A is a member of cells cycle protein and implicated in the control of DNA replication. It is linked to both CDK 1 (cyclin dependent kinase) and CDK2, and has functions in both S phase and mitosis stages of the cell cycle [126]. High expression of cyclin A is observed in various human malignancies like astrocytoma, breast cancer, and liver cancer [127]. TOP2A (topoisomerase II α) is an essential cellular enzyme involved in modification of DNA topology for maintaining the functioning of DNA replication [128, 129]. Our meta-analysis revealed the association of the high expression of cyclin A and TOP2A with the high-risk of recurrence of meningioma patients. These markers could be used as predictors of recurrence for meningioma patients. Abnormal expression of cyclin A and TOP2A may linked to the irregular cell proliferation of meningioma patients.

p21 (also called p21^WAF1/Cip1^) is a small protein with 165 amino acids and belongs to the CIP/Kip family of CDKs inhibitors [130]. It arrests the cell cycle progression in G1/S and G2/M transitions by inhibiting CDK4,6/cyclin-D and CDK2/cyclin-E [131]. Recent meta-analysis reported that low p21 expression was an unfavorable prognostic biomarker in patients with esophageous cancer [132]. This result is consistent with our finding that low p21 expression was associated with poor prognosis of meningioma patients. The minichromosome maintenance 6 (MCM6) functions as a regulator of DNA licensing, and plays a key role in cell cycle progression [133]. Overexpression of MCM6 may predict the unfavorable survival outcomes of patients with glioma [134], hepatocellular carcinoma (HCC) [135] and endometrioid endometrial adenocarcinoma [136]. Our meta-analysis identified that high expression of MCM6 was not associated with PFS of meningioma patients. Further studies on large patient cohorts are needed to evaluate the prognostic value of MCM6 in meningioma patients.

Histone modifications, including acetylation and methylation, are essential epigenetic mechanisms involved in the regulation of gene expression and have been implicated in cancer development [137, 138]. One specific histone modification, trimethylation of lysine 27 (K27) of histone H3 (H3K27me3), plays a significant role in tumorigenesis of various cancers like meningioma, glioma, and breast cancer [30, 139–142]. Recent meta-analysis has revealed that loss of H3K27me3 was associated with poor RFS but not associated with OS of meningioma patients [143]. However, in our study, no significant association was observed between H3K27me3 expression and prognosis of meningioma patients measured by OS, RFS, and PFS of meningioma patients. This may because of the difference of the studies pooled in the analysis. Moreover, several other factors might influence the prognostic significance of H3K27me3, such as discrepancies between immunohistochemistry evaluation and theoretically defined loss of H3K27me3 and different histological grade of meningioma. Furthermore, the inclusion of studies, with inadequate patient follow-up time might affect the prognosis of meningioma patients [143]. Therefore, more comprehensive and extensive research should be conducted to clarify this issue.

Bcl-2 (B cell lymphoma 2) is the member of the Bcl-2 family of regulatory proteins that regulate cell death (apoptosis), either by promoting or inhibiting apoptotic cell death [144, 145]. Bcl-2 is initially identified as an oncogene in cases of human follicular B-cell lymphoma with a t(14;18) chromosome translocation [146, 147]. It has also been linked to various types of tumors such as lung cancer, breast cancer, gastric cancer, non-small cell lung cancer, and head and neck cancer [148–152]. Our results showed no association between Bcl-2 expression and recurrence of meningioma patients. In the future, it might be necessary to confirm the Bcl-2 expression in large cohorts of patients.

p53 is the most frequently affected gene in human cancer. It has a dual role in the regulation of cell proliferation and apoptosis either arresting cells at G1 phase to allow replication of damaged DNA or inducing apoptosis when DNA damage is irreversible [153]. When p53 have lost functioning, tumor cells may resist cell-cycle arrest and apoptosis and continue to proliferate abnormal cells [153]. In our study, p53 emerged as a valuable biomarker for RFS of meningioma patients. However, p53 overexpression was not associated with OS in meningioma patients. Prognostic value of p53 was already reported in hepatocellular carcinoma and upper urinary tract urothelial carcinoma [154, 155], but no prognostics value in astrocytoma and Hodgkin’s lymphoma [156, 157]. VEGF (vascular endothelial growth factor), a glycoprotein with a molecular weight of approximately 45 kDa, accelerate the cell invasion, metastasis, and disease progression by participating in angiogenesis [158, 159]. Several systematic reviews and meta-analyses have investigated the predictive significance of VEGF in various types of cancer, including head and neck squamous cancer, lung cancer, colon cancer, gastric cancer, and hepatocellular carcinoma [160–164]. They demonstrated a correlation between increased expression of VEGF and unfavorable outcomes. Moreover, it was reported high VEGF level in glioma, suggesting that this could be a potential marker for these patients [165]. Our study identified that high VEGF expression was associated with poor RFS of meningioma patients.

The correlation between increased cellular proliferation and shorter survival time of patients is observed in various cancers [166–168]. Mitotic index (MI) counted by phosphorylation of histone H3 (PHH3) is a mitosis specific marker involving in the initiation of mitosis during early prophase of the cell cycle and is absent during other phases of cell cycle and chromatin changes [169–171]. Two recent meta-analyses studies revealed that MI assessed through both H&E and PHH3 staining could be a prognostic marker of atypical meningioma patients [172, 173]. However, we showed that PHH3 MI was not associate with RFS of meningioma patients of all WHO grades. The MI is variable within samples, leading to discrepancies among pathologists and even among different readings. Additionally, the variability in stain selection, quality, and intensity further contributes to the heterogeneity of this parameter [88].

Ki-67/MIB-1 is a non-histone nuclear protein that is expressed throughout the active phases of cells cycle (G1, S, G2, and M) and mitosis [174]. Several studies identified the expression of Ki-67 as the prognostic biomarker of various cancers such as meningioma, glioma, renal cells carcinoma, thyroid cancer, prostate cancer, and bladder cancer [12, 175–179]. Previous meta-analysis revealed that Ki-67 was a prognostic biomarker correlating with OS and RFS, PFS and DFS of meningioma patients [12]. In this study, we evaluated the prognostic value of Ki-67 for OS, RFS, and PFS of meningioma patients using meta-analysis. Our results showed that high Ki-67 expression was associated with poor OS, RFS and PFS. In our meta-analysis study, we measured the published HR value of each study in meta-analysis. In the previous study, they measured not only published HR but also the HR using several time points on the graphical representation of the survival curves. However, the estimated HR might be less reliable than when obtained from published statistics. In this study, we used the cut-off 4% as a threshold based on the literature review [180]. we found the significant heterogeneity in Ki-67 cut-off >4 in OS, RFS, and PFS. The heterogeneity was also related to low and high WHO grade of meningioma patients. Our study revealed that there was a significant publication bias in the analysis of the prognostic value of Ki-67 using OS. This result was similar to the previous study on the prognostic value of Ki-67 for meningioma patients. They found a significant bias after refilling studies using Trim and Fill method in the publication bias assessments [12]. Thus, considerable caution is required for the evaluation of Ki-67 overexpression of meningioma patients.

Molecular profiling has highlighted the benefits of using genetic and epigenetic alterations to further determine the aggressiveness and recurrence risk of meningioma [181, 182]. Multiple studies have demonstrated that the presence of TERT promoter mutation in meningiomas correlates with a poorer prognosis and reduced OS regardless of the WHO grades [102, 183–186]. In addition, CDKN2A/B status is considered an independent prognostic factor in meningioma [187, 188]. Furthermore, three CDKN2A alteration (p.Ala148Thr) mutation, entire homozygous or heterozygous gene deletion, or promotor methylation (>8) were significantly associated with recurrence and Ki67 labeling index [187].

The strength of this study is the systematic review and meta-analysis on the association between immunohistochemically detectable biomarkers and the prognosis of meningioma patients. Extensive literature search was conducted with constraints to exclude related studies. The analysis was carried out on the association of the degree of the expression of immunohistochemical biomarkers and prognosis of meningioma patients. Several limitations of the present study must be acknowledged. First of all, in most of the studies, WHO grade I, II, and III meningioma were not separately evaluated. Biological behaviors of meningiomas varies not only among different grades but also even within a each grade [189]. Grade I meningiomas also have clinical outcomes that are similar to those of grade II [190]. Grade II tumors have a wide range of histological features and behaving biologically like grades I or III tumors [60, 191]. The chance of the recurrence is up to 25% in grade I meningiomas, 59% in grade II, and up to 94% in grade III [9]. We are unable to see whether there is a biological difference among the markers which affect on the prognosis of varying grades of meningioma. Second, IHC was used to measure the degree of the expression of biomarkers in all studies included, but the cut-off points or reference groups used were variable among the studies, which may potentially contribute to the heterogeneity. For example, some studies used no stain as reference group (negatives), where others used tumors with 5% or 10% positive cells as a reference. The criteria to determine the expression level of biomarkers were inconsistent among the studies. In addition to IHC, other methods, such as RT-PCR, and fluorescence in situ hybridization (FISH) should be considered to identify prognostic biomarkers. More comprehensive search on the standard and methods for prognostic biomarkers for meningioma patients should be defined in the future. Third, a significant heterogeneity was observed in certain biomarkers analysis, probably due to differences in factors such as patient’s gender, age, tumor stage and grade and variations in cut-off values used for the biomarkers. Fourth, we were unable to determine a summary HR from meta-analyses, because 33 biomarkers (about 75%) had sufficient data available only from a single study. Hence prognosis markers should be confirmed in further investigations. Fifth, most of the studies were retrospective design, with small sample sizes, and conducted meta-analyses which may affect the statistical power of the analyses. Approximately 24% of the studies included showed a high risk of bias mainly due to inadequate control of cofounding factors, such as tumor grade, tumor status and tumor resection. Thus, further studies are required for the better addressing of these aspects. Finally, studies without HRs were not included in the final assessment. Therefore, more well-designed and large-scale prospective studies are needed to confirm our findings.

## Conclusion

In conclusion, our study identified several prognostic biomarkers for meningioma patients, including PR, cyclin A, TOP2A, p21, p53, VEGF and Ki-67. By incorporating these biomarkers, the prognostic evaluation of meningioma patients might be improved, leading to better survival outcomes and aiding in personalized therapy decisions for individuals with meningioma.

## Supporting information

S1 File PRISMA 2020 checklist. (PDF)

S1 Fig Publication bias determined by funnel plot analysis for the prognostic value of Ki-67 using OS. (TIF)

S1 Table Search strategy. (DOCX)

S2 Table Demographic characteristics of included studies. (DOCX)

S3 Table Quality assessments of included studies using QUIPS six domains. (DOCX)

S4 Table Subgroup analysis of cyclin A on recurrence-free survival of meningioma patients. (DOCX)

S5 Table Subgroup analysis of H3K27me3 overall survival, recurrence-free survival and progression-free survival of meningioma patients. (DOCX)

S6 Table Subgroup analysis of PHH3 on recurrence-free survival of meningioma patients. (DOCX)

S7 Table Subgroup analysis of ki67 on overall survival, recurrence-free survival and progression-free survival of meningioma patients. (DOCX)
