# Supplementary figures and images for: Biomarkers for prognosis of meningioma patients: A systematic review and meta-analysis
Source: PLoS One. 2024 May 17;19(5):e0303337. doi: 10.1371/journal.pone.0303337 (PMC11101050; doi:10.1371/journal.pone.0303337)

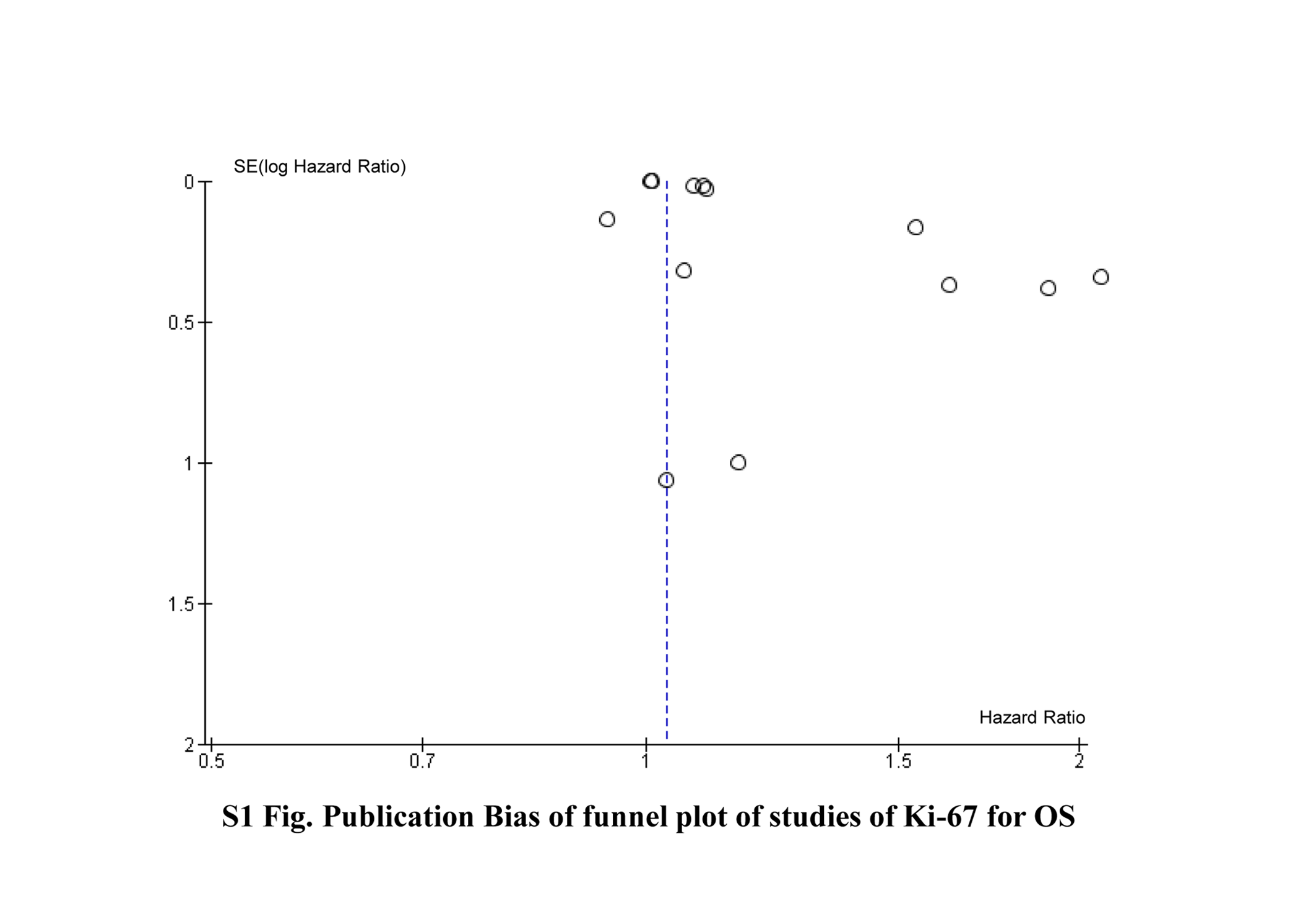

Supplement: S1 Fig — (TIF) [file pone.0303337.s002.tif]
