# Supplementary material for: Biomarkers for prognosis of meningioma patients: A systematic review and meta-analysis
Source: PLoS One. 2024 May 17;19(5):e0303337. doi: 10.1371/journal.pone.0303337 (PMC11101050; doi:10.1371/journal.pone.0303337)
Supplement: S1 Table — (DOCX) [file pone.0303337.s003.docx]

**S1 Table. Search strategy**

| **PubMed :** https://pubmed.ncbi.nlm.nih.gov/ | | **Results** |
| --- | --- | --- |
| 1 | ("Meningioma"[Mesh] OR "Meningeal Neoplasms"[Mesh]) OR (meningioma[Title/Abstract] OR Meningeal Neoplasms[Title/Abstract] OR meningeal tumor[Title/Abstract] OR brain tumor[Title/Abstract]) | 58,024 |
| 2 | ("Biomarkers"[Mesh] OR "Biomarkers, Tumor"[Mesh] OR "Blood Chemical Analysis"[Mesh] OR "Blood Chemical Analysis/diagnosis"[Mesh] OR "Immunohistochemistry"[Mesh]) OR (biomarker[Title/Abstract] OR Immunohistochemistry[Title/Abstract] OR blood assay*[Title/Abstract]) | 1,762,621 |
| 3 | ("Prognosis"[Mesh] OR "Early Diagnosis"[Mesh]) OR (Prognosis[Title/Abstract] OR predict*[Title/Abstract] OR Diagnos*[Title/Abstract]) | 6,417,691 |
| 4 | ("Survival"[Mesh] OR "Mortality"[Mesh] OR "mortality" [Subheading] OR "Survival Rate"[Mesh] OR "Recurrence"[Mesh] OR "Neoplasm Recurrence, Local"[Mesh]) | 1,148,835 |
| 5 | ("Case-Control Studies"[Mesh] OR "Cohort Studies"[Mesh] OR "Cross-Sectional Studies"[Mesh]) OR ( "Randomized Controlled Trials as Topic"[Mesh] OR "Randomized Controlled Trial" [Publication Type] OR "Controlled Clinical Trials as Topic"[Mesh] OR "Non-Randomized Controlled Trials as Topic"[Mesh] ) | 3,757,003 |
| 6 | "Humans"[Mesh] | 21,572,114 |
| 7 | (#1 AND #2) AND (#3 AND #4) | 722 |
| 8 | (#5 AND #6) AND #7 | 237 |
| 9 | "Systematic Review" [Publication Type] OR "Systematic Reviews as Topic"[Mesh] | 255,505 |
| 10 | #8 NOT #9 | 234 |

| **CENTRAL :** https://www.cochranelibrary.com/ | | **Results** |
| --- | --- | --- |
| 1 | MeSH descriptor: [Meningioma] explode all trees | 111 |
| 2 | MeSH descriptor: [Meningeal Neoplasms] explode all trees | 174 |
| 3 | (meningioma):ti,ab,kw OR (Meningeal Neoplasm*):ti,ab,kw OR (meningeal tumor):ti,ab,kw OR (brain tumor):ti,ab,kw | 5,226 |
| 4 | #1 OR #2 OR #3 | 5, 228 |
| 5 | MeSH descriptor: [Biomarkers] explode all trees | 26,992 |
| 6 | MeSH descriptor: [Biomarkers, Tumor] explode all trees | 6,535 |
| 7 | MeSH descriptor: [Blood Chemical Analysis] explode all trees | 8,504 |
| 8 | MeSH descriptor: [Immunohistochemistry] explode all trees | 5,634 |
| 9 | (biomarker*):ti,ab,kw OR ("blood chemical analysis"):ti,ab,kw OR (immunohistochemistry):ti,ab,kw | 58,177 |
| 10 | #5 OR #6 OR #7 OR #8 OR #9 | 73,543 |
| 11 | #4 AND #10 | 588 |
| 12 | MeSH descriptor: [Prognosis] explode all trees | 203,795 |
| 13 | MeSH descriptor: [Early Diagnosis] explode all trees | 2,859 |
| 14 | (Prognosis):ti,ab,kw OR (Predict*):ti,ab,kw OR (Diagnos*):ti,ab,kw | 408,861 |
| 15 | #12 OR #13 OR #14 | 532,278 |
| 16 | #11 AND #15 | 392 |
| 17 | MeSH descriptor: [Survival] explode all trees | 2,879 |
| 18 | MeSH descriptor: [Mortality] explode all trees | 21,892 |
| 19 | MeSH descriptor: [Survival Rate] explode all trees | 13,367 |
| 20 | MeSH descriptor: [Recurrence] explode all trees | 14,647 |
| 21 | MeSH descriptor: [Neoplasm Recurrence, Local] explode all trees | 6,274 |
| 22 | #17 OR #18 OR #19 OR #20 OR #21 | 43,078 |
| 23 | #16 AND #22 in Trials | 59 |

| **CINAHL Plus :** https://web.s.ebscohost.com/ | | **Results** |
| --- | --- | --- |
| 1 | TI meningioma OR AB meningioma OR TI neoplasms OR AB neoplasms OR TI meningeal neoplasms OR AB meningeal neoplasms OR TI meningeal tumor OR AB meningeal tumor OR TI brain tumor OR AB brain tumor  Expanders - Apply equivalent subjects  Search modes - Find all my search terms | 238,185 |
| 2 | TI biomarkers OR AB biomarkers OR TI biomarkers, tumor OR AB biomarkers, tumor OR TI blood chemical analysis OR AB blood chemical analysis OR TI immunohistochemistry OR AB immunohistochemistry  Expanders - Apply equivalent subjects  Search modes - Find all my search terms | 99,312 |
| 3 | TI prognosis OR AB prognosis OR TI early diagnosis OR AB early diagnosis OR TI diagnos* OR AB diagnos* OR TI predict* OR AB predict*  Expanders - Apply equivalent subjects  Search modes - Find all my search terms | 1,094,102 |
| 4 | TI survival OR AB survival OR TI mortality OR AB mortality OR TI recurrence OR AB recurrence OR TI Neoplasm Recurrence OR AB Neoplasm Recurrence  Expanders - Apply equivalent subjects  Search modes - Find all my search terms | 458,401 |
| 5 | S1 AND S2 AND S3 AND S4  Expanders - Apply equivalent subjects  Search modes - Find all my search terms | 7,580 |
| 6 | TI case control study OR AB case control study OR TI cohort study OR AB cohort study OR TI cross sectional study OR AB cross sectional study OR TI ( randomized controlled trials or rtc or randomised control trials ) OR AB ( randomized controlled trials or rtc or randomised control trials ) OR TI non-random* controlled trials OR AB non-random* controlled trials OR TI controlled clinical trial OR AB controlled clinical trial  Expanders - Apply equivalent subjects  Search modes - Find all my search terms | 621,494 |
| 7 | S5 AND S6  Limiters - Human  Expanders - Apply equivalent subjects  Search modes - Find all my search terms | 912 |

| **Scopus** : https://www.scopus.com/ | **Results** |
| --- | --- |
| ( ( ( ( TITLE-ABS-KEY ( meningioma )  OR TITLE-ABS-KEY ( meningeal  AND neoplasms )  OR  TITLE-ABS-KEY ( meningeal  AND tumor )  OR  TITLE-ABS-KEY ( brain  AND tumor ) ) )  AND  ( ( TITLE-ABS-KEY ( biomarker* )  OR  TITLE-ABS-KEY ( blood  AND chemical  AND analysis )  OR  TITLE-ABS-KEY ( immunohistochemistry )  OR  TITLE-ABS-KEY ( blood  AND assay* ) ) )  AND  ( ( TITLE-ABS-KEY ( prognosis )  OR  TITLE-ABS-KEY ( early  AND diagnosis )  OR  TITLE-ABS-KEY ( predict* )  OR  TITLE-ABS-KEY ( diagnos* ) ) ) )  AND  ( ( TITLE-ABS-KEY ( survival )  OR  TITLE-ABS-KEY ( mortality )  OR  TITLE-ABS-KEY ( recurrence )  OR  TITLE-ABS-KEY ( neoplasm  AND recurrence ) ) )  AND  ( TITLE-ABS-KEY ( humans ) ) )  AND  ( ( TITLE-ABS-KEY ( case-control  AND study )  OR  TITLE-ABS-KEY ( cohort  AND study )  OR  TITLE-ABS-KEY ( cross-sectional  AND study )  OR  TITLE-ABS-KEY ( random*  AND control*  AND trial )  OR  TITLE-ABS-KEY ( controlled  AND clinical  AND trial )  OR  TITLE-ABS-KEY ( non-random*  AND control*  AND trial ) ) )  AND  ( LIMIT-TO ( DOCTYPE ,  "ar" ) )  AND  ( LIMIT-TO ( SRCTYPE ,  "j" )  OR  LIMIT-TO ( SRCTYPE ,  "p" ) ) | 2,251 |
