# Supplementary material for: Biomarkers for prognosis of meningioma patients: A systematic review and meta-analysis
Source: PLoS One. 2024 May 17;19(5):e0303337. doi: 10.1371/journal.pone.0303337 (PMC11101050; doi:10.1371/journal.pone.0303337)
Supplement: S2 Table — (DOCX) [file pone.0303337.s004.docx]

**S2 Table. Demographic characteristics of included studies**

| **Study** | **Biomarkers** | **Year of recruitment** | **Country** | **Sample size** | **Age range (years)** | **Female (%)** | **WHO grade** | **Method** | **Outcomes** | **Maximum follow-up** |
| --- | --- | --- | --- | --- | --- | --- | --- | --- | --- | --- |
| Abdelzaher, et al., 2011 | Ki-67, PR, p53, Bcl2, Her2 | 2004 - 2009 | Egypt | 60 | 6 - 79 | 75 | GI | IHC | RFS | 66 months |
| Abdelzaher, et al., 2013 | VEGF, PR | 2004 - 2013 | Egypt | 265 | 12 - 80 | 58 | Meningioma | TMA IHC | RFS | 100 months |
| Ammendola, et al., 2022 | H3K27me3 | NA | Italy | 39 | 35 - 86 | 33 | GI, GII | IHC | PFS | 143 months |
| Anand, et al., 2022 | Ki-67 | 2010 - 2019 | Iran | 103 | mean 51.1 | 66 | GII | IHC | RFS | 118 months |
| Assimakopoulou, et al., 2023 | Polycystin-2 | NA | Greek | 82 | mean 60.06 | 74 | GI, GII, GIII | IHC | OS | 124 months |
| Barresi, et al., 2006 | Ki-67, Cav-1 | 1996 -1998 | Italy | 62 | 21 - 84 | 60 | GI, GII | IHC | OS | 120 months |
| Barresi, et al., 2015 | p-CREB, Ki-67 | 2003 - 2011 | Italy | 47 | mean 62 | NA | GI, GII | IHC | DFS | 120 months |
| Baumgarten, et al., 2016 | Ki-67 | NA | Germany | 229 | NA | NA | GII | IHC | PFS | 202 months |
| Behling, et al., 2021 | H3K27me3, MIB-1 | 2003 - 2005 | Germany | 1,268 | 8.3 - 91 | 72 | GI, GII, GIII | TMA IHC | RFS | 186.3 months |
| Behling, et al., 2023 | S100, MIB-1 | 2003 – 2017 | Germany | 1,669 | NA | 72 | GI, GII, GIII | TMA IHC | RFS | 195.6 months |
| Bruna, et al., 2007 | Ki-67 | 1980 - 2001 | Spain | 28 | 16 - 80 | 43 | GII, GIII | IHC | OS, RFS | 282.8 months |
| Cai, et al., 2017 | Ki-67, TXNIP | 2003 - 2010 | China | 65 | 28 - 74 | 58 | GI, GII, GIII | IHC | RFS | 84 months |
| Cardona, et al., 2019 | VEGFR2, PDGFRβ | 2011 – 2016 | Colombia | 31 | 28 – 88 | 71 | GII, GIII | IHC | OS, PFS | median 31.8 months |
| Champeaux, et al., 2016 | Ki-67 | 2000 - 2015 | UK | 178 | 44.7 – 68.8 | 54 | GIII | IHC | RFS | median 3.6 years |
| Champeaux, et al., 2017 | Ki-67 | 2000 - 2016 | UK | 215 | median 56.9 | 55 | GII | IHC | RFS | 22.97 years |
| Chang, et al., 2023 | Ki-67 | 1998 – 2018 | Korea | 523 | NA | 61 | GII | IHC | PFS | median 45 months |
| Chen, et al., 2021 | Ki-67 | 2011 - 2019 | China | 128 | 33 | 62 | Atypical meningioma | IHC | RFS | 77 months |
| Choi, et al., 2018 | Ki-67 | 2001 - 2012 | Korea | 50 | 13 -78 | 48 | GII | IHC | RFS | median 47.4 months |
| Damen, et al., 2021 | Ki-67 | 2002 – 2015 | Netherland | 44 | NA | 70 | GI | IHC | RFS | 137 months |
| Deguchi, et al., 2020 | Ki-67 | 2003 – 2016 | Japan | 124 | 32 – 84 | 73 | GI | IHC | RFS | 180 months |
| Di Bonaventura, et al., 2022 | SOX2 | 2004 - 2019 | Italy | 87 | median 61 | 48 | GI, GII, GIII | IHC | OS, PFS | 122.9 months |
| Endo, et al., 2016 | MIB-1 | 2000 - 2013 | Japan | 45 | 5 - 82 | 44 | GII | IHC | OS, RFS | 187 months |
| Gauchotteet, et al., 2017 | Ki-67, MCM6, Cyclin D1 | 1997 - 2013 | France | 85 | 29 - 85 | 54 | GI, GII, GIII | IHC | OS, PFS | 96 months |
| Gauchotteet, et al., 2020 | H3K27me3 | 1990 - 2015 | France | 66 | 21 - 86 | 52 | GIII | IHC | OS, RFS | 287.6 months |
| **Study** | **Biomarkers** | **Year of recruitment** | **Country** | **Sample size** | **Age range (years)** | **Female (%)** | **WHO grade** | **Method** | **Outcomes** | **Maximum follow-up** |
| Gauchotteet, et al., 2023 | MCM6, Ki-67 | 2001 – 2018 | France | 169 | 29 – 91 | 72 | GI, GII | IHC | OS, PFS | NA |
| Gousias, et al., 2014 | KPNA2, CRM1 | 1996 - 2008 | Germany | 108 | 25 - 80 | 59 | GI, GII, GIII | IHC | PFS | median 12 years |
| Guadagno, et al., 2016 | Ki-67, PR, p40 | 2004 - 2008 | Italy | 72 | 21 - 80 | 69 | GI, GII | IHC | OS, RFS | 120 months |
| Guillaudeau, et al., 2012 | Ki-67, EGFR ECD Ab, EGFR ICD Ab | 1995 - 2009 | France | 69 | median 56.8 | 62 | GI, GII, GIII | IHC | OS, PFS | NA |
| Han, et al., 2016 | CD20^+^, PDL^+^/CD68^-^ | 1989 - 2012 | USA | 96 | 18.7 -86.5 | 59 | GI, GII, GIII | TMA IHC | OS, PFS | NA |
| Hsu, et al., 1998 | MIB-1, TGF-α | 1971 - 1992 | USA | 57 | 15 - 78 | 63 | GI, GII, GIII | IHC | PFS | mean 81.9 months |
| Hua, et al., 2017 | ER, PR, Ki-67 | 2003 - 2008 | China | 87 | 6 - 79 | 46 | GIII | IHC | OS, PFS | 160 months |
| Hua, et al., 2020 | H3K27me3, Ki-67 | NA | China | 192 | 18 - 80 | 57 | GI, GII, GIII | IHC | OS, PFS | 209 months |
| Hua, et al., 2023 | H3K27me3, Ki-67 | 2009 – 2013 | China | 164 | NA | 54 | GI, GII | TMA IHC | OS, PFS | 156 months |
| Jensen, et al., 2012 | MIB-1, VEGF, HIF 1α, GLUT-1, CA-IX | NA | Utah | 263 | 21 - 95 | 66 | GI, GII, GIII | IHC | OS, PFS | median 75.3 months |
| Jiang, et al., 2012 | UbcH10, Ki-67 | 2002 - 2009 | China | 47 | median 51 | 57 | GI, GII,GIII | IHC | RFS | 80 months |
| Jung, et al., 2021 | H3K27me3, Ki-67, PHH3 | 2010 - 2019 | Korea | 141 | 17 - 90 | 57 | GII, GIII | TMA IHC | OS, RFS | NA |
| Kalala, et al., 2004 | Ki-67, PCNA | 1986 - 1997 | Belgium | 125 | 7 - 84 | 73 | GI, GII, GIII | IHC | RFS | NA |
| Karsy, et al., 2018 | Ki-67 | 1996 - 2011 | Canada | 207 | NA | 70 | GI | IHC | PFS | NA |
| Katz, et al., 2018 | H3K27me3 | NA | Germany | 232 | NA | 53 | GI, GII, GIII | IHC | RFS | NA |
| ke, et al., 2014 | LASS2, Ki-67, p53 | 2000 - 2005 | China | 143 | 8 - 79 | 55 | GI, GII, GIII | IHC | OS | 121 months |
| Kim, et al., 2006 | Ki-67 | 1997 - 2002 | Germany | 181 | NA | NA | GI, GII, GIII | IHC | RFS | 96 months |
| Kim, et al., 2007 | Ki-67, PHH3 | 1996 - 2002 | Germany | 99 | NA | NA | GI, GII, GIII | IHC | RFS | 120 months |
| Kim, et al., 2012 | MIB-1 | 1998 - 2010 | Korea | 35 | 12 - 82 | 54 | GII, GIII | IHC | RFS | 107 months |
| Kim, et al., 2014 | p16, p21, CDK4, CDK6, pRB, Cyclin D1, p53, MIB-1, p15, p17 | 2000 - 2012 | Korea | 67 | 26.4 - 87.2 | 58 | GII | IHC | RFS | 132.1 months |
| Kim, et al., 2018 | Ki-67 | 2007 - 2014 | Boston | 76 | NA | 53 | GII, GIII | IHC | OS, PFS | median 52.6 months |
| Klinger, et al., 2015 | MIB-1 | 2000 - 2012 | USA | 57 | mean 57.6 | 58 | GII | IHC | RFS | 120 months |
| Konstantinidou, et al., 2003 | PR, ER, AR, Ki-67 | 1998 - 1995 | Greece | 51 | NA | 61 | GI, GII, GIII | IHC | DFS | 108 months |
| Korshunov, et al., 2002 | Ki-67, Topoisomerase II α, Cyclin A | 1993 - 1996 | Russia | 263 | median 48 | 75 | GI, GII, GIII | IHC | RFS | 92 months |
| **Study** | **Biomarkers** | **Year of recruitment** | **Country** | **Sample size** | **Age range (years)** | **Female (%)** | **WHO grade** | **Method** | **Outcomes** | **Maximum follow-up** |
| Korshunov, et al., 2003 | Topoisomerase II α, p16, p18, p21, p14, p27, p73 | NA | Russia | 271 | median 49 | 74 | GI, GII, GIII | IHC | RFS | 92 months |
| Koschny, et al., 2015 | RTRAIL-R2, RTRAIL-R4, Caspase-8, cFLIP, Bak, Bcl-XL, Mcl-1 | 2008 - 2011 | Germany | 37 | 25 - 81.5 | 62 | GI, GII, GIII | IHC | OS, PFS | NA |
| Kuo, et al., 2019 | Beclin, LC3B | 2005 - 2008 | Taiwan | 77 | NA | 58 | GI, GII, GIII | IHC | OS | NA |
| Li, et al., 2016 | PR, Ki-67 | 2008 - 2015 | China | 36 | 4 - 77 | 64 | Clear cell meningioma | IHC | PFS | 5 years |
| Ling, et al., 2016 | Ki-67 | 1997 - 2001 | France | 139 | 33 - 90 | 66 | GI, GII, GIII | IHC | OS, PFS | 4889 days |
| Liu, et al., 2017 | Ki-67 | 2005 - 2014 | USA | 59 | 21 - 81 | 44 | GII | IHC | OS, RFS | NA |
| Liu, et al., 2021 | SP1 | NA | Taiwan | 74 | Mean 58.05 | 57 | GI, GII, GIII | IHC | RFS | NA |
| Maier, et al., 2020 | Ki-67, PHH3 | 2000 - 2014 | Denmark | 24 | 31 - 88 | 54 | GIII | IHC | PFS | 282 months |
| Marciscano, et al., 2016 | MIB-1 | 2002 - 2012 | Massachusetts | 148 | 13 - 88 | 79 | GI | IHC | PFS | median 37.5 months |
| Matsuno, et al., 1996 | MIB-1 | 1986 - 1992 | Japan | 127 | mean 50.9 | 39 | Meningioma | IHC | RFS | > 3 years |
| Mirian, et al., 2020 | Ki-67 | 2005 – 2008 | Denmark | 159 | 19 -88 | 79 | GI, GII, GIII | IHC | RFS |  |
| Moutafidi, et al., 2021 | HST1, TRPV1, TRPA1 | NA | Greece | 71 | 31 - 87 | NA | GI, GII | IHC | OS | 124 months |
| Nakabayashi, et al., 2003 | Ki-67, Cyclin A | 1989 - 1994 | Japan | 77 | 32 - 72 | 62 | GI, GII, GIII | IHC | RFS | 7 years |
| Nakasu, et al., 2009 | MIB-1 | 1982 - 2004 | Japan | 135 | NA | NA | GI | IHC | RFS | 21 years |
| Nakya, et al., 2009 | MIB-1 | 1995 - 2005 | Japan | 34 | 31 - 72 | 71 | GI, GII, GIII | IHC | PFS | 132 months |
| Nanda, et al., 2016 | Ki-67 | 1995 - 2014 | USA | 59 | 21 - 88 | 42 | GII | IHC | RFS | 195 months |
| Nassiri, et al., 2021 | H3K27me3 | 2000 - 2017 | USA | 151 | 44 - 66 | 60 | GI, GII, GIII | IHC | RFS | NA |
| Nowak-Choi, et al., 2021 | Ki-67 | 2005 - 2013 | USA | 299 | Mean 59.5 | 69 | GI | IHC | RFS | 123.7 months |
| Ohba, et al., 2011 | MIB-1, p53 | 1980 - 2004 | Japan | 266 | 6 - 81 | 80 | GI, GII, GIII | IHC | PFS | median 76 months |
| Olar, et al., 2015 | Ki-67, PHH3 | NA | USA | 363 | NA | NA | GI, GII, GIII | IHC | RFS | NA |
| Olar, et al., 2017 | PHH3 | NA | USA | 137 | NA | 42 | GI | IHC | RFS | NA |
| Oya, et al., 2012 | MIB-1 | 1995 - 2010 | Japan | 248 | 12 - 80 | 73 | GI | IHC | RFS | NA |
| Parada, et al., 2018 | AKAP12 | 1998 - 2012 | USA | 75 | NA | NA | GI, GII, GIII | TMA IHC | PFS | NA |
| Parada, et al., 2020 | RB1 S780phosphorylation | 1998 - 2012 | Washington | 140 | NA | NA | GI, GII, GIII | TMA IHC | PFS | 120 months |
| Park, et al., 2022 | Ki-67 | 2005 – 2019 | Korea | 39 | mean 52 | 79 | GI | TMA IHC | RFS | mean 88 months |
| Perry, et al., 1998 | MIB-1, p53 | 1978 - 1988 | Minnesota | 425 | 15 - 93 | 66 | Meningioma | IHC | RFS | NA |
| **Study** | **Biomarkers** | **Year of recruitment** | **Country** | **Sample size** | **Age range (years)** | **Female (%)** | **WHO grade** | **Method** | **Outcomes** | **Maximum follow-up** |
| Potti, et al., 2004 | Her2 | 1986 - 1999 | North Dakota | 188 | 0 - 93 | 53 | Meningioma | IHC | OS | NA |
| Prat-Acin, et al., 2021 | Ki-67 | NA | Spain | 365 | NA | 70 | GI, GII | IHC | OS | 120 months |
| Ren, et al., 2022 | Ki-67, PR | 2010 – 2018 | China | 231 | 18 – 25 | 48 | Atypical meningioma | IHC | PFS | 152.5 months |
| Roser, et al., 2004 | Ki-67 | 1990 - 2000 | Germany | 554 | 15 - 94 | 67 | GI, GII, GIII | IHC | RFS | 228 months |
| Samal, et al., 2020 | H3K27me3, EZH2, DNMT-1, DNMT-3A, DNMT-3B | 2015 - 2018 | India | 149 | 11 - 85 | NA | GI, GII | IHC | PFS | 258.4 weeks |
| Sanz, et al., 2013 | Cox-2, Ki-67, TIMP-2, Topoisomerase II α, Cyclin A, pAKT, Bcl-2, Cadherin E, Caspase 3a, beta-catenin, Cathepsin D, CD44, EGFR, Her2, MDM2, MMP9, p21, PDGF, PTEN, PR, Survivin, Btea-TGF, Alpha-VEGF | 1980 - 2005 | Spain | 135 | 24 - 82 | 70 | GI, GII | TMA IHC | RFS | 25 years |
| Shan, et al., 2017 | Ki-67 | 2008 - 2016 | China | 42 | 20 - 80 | 52 | GIII | IHC | OS | 75 months |
| Sun, et al., 2020 | Ki-67 | 2012 - 2016 | China | 1,107 | 13 - 85 | 71 | GI, GII, GIII | IHC | RFS | NA |
| Toland, et al., 2020 | H3K27me3 | 1989 - 2017 | Columbus | 46 | 2 - 18 | 48 | GI, GII, GIII | IHC | RFS | 282 months |
| Tsai, et al., 2016 | Nrf2 | 1991 - 2005 | Japan | 69 | NA | NA | GI, GII, GIII | TMA IHC | OS | 5 years |
| Tsai, et al., 2018 | DDX3X | NA | Taiwan | 71 | NA | NA | GI, GII | TMA IHC | OS | 5 years |
| Ulgen, et al., 2019 | Ki-67, ER^+^PR^+^S100^-^ | 2003 - 2016 | Turkey | 366 | 18 - 100 | 18 | GI, GII, GIII | IHC | OS, RFS | 144 months |
| Vaubel, et al., 2023 | H3K27me3 | 1988 – 2011 | USA | 63 | 27.4 – 90.6 | 65 | GII | IHC | RFS | 24.1 years |
| Vranic, et al., 2010 | Ki-67 | 1990 - 2005 | Slovenia | 86 | 13.5 - 85.4 | 51 | GII, GIII | IHC | OS, RFS | 204 months |
| Wang, et al., 2012 | MIB-1 | 1997 - 2010 | China | 123 | <18 | NA | GI, GII, GIII | IHC | OS, PFS | 177 months |
| Wang, et al., 2013 | MIB-1, PR^-^Bcl-2^+^ | 1997 - 2011 | China | 30 | 5 - 72 | 47 | Papillary meningioma | IHC | PFS | 197 months |
| Wang, et al., 2014 | AK2, Ki-67 | 1998 - 2010 | China | 94 | NA | 60 | GI, GII, GIII | IHC | RFS | NA |
| Winther, et al., 2016 | PHH3, Ki-67 | 1991 - 2000 | Norway | 160 | 25 - 86 | 75 | GI, GII | TMA IHC + IHC | RFS | NA |
| Winther, et al., 2017 | MCM7 | 1998 - 2000 | Norway | 160 | 25 - 86 | 75 | GI, GII | TMA | OS | NA |
| Yamaguchi, et al., 2014 | MIB-1 | 1995 | Japan | 55 | 23 - 84 | 51 | GII, GIII | IHC | RFS | median 43.9 months |
| Yamamoto, et al., 2021 | hENT1,dCK | 1996 - 2019 | Japan | 45 | NA | NA | GI, GII, GIII | IHC | PFS | NA |
| Yamasaki, et al., 2000 | VEGF, MIB-1 | 1970 - 1999 | Japan | 54 | 28 - 77 | 70 | GI | IHC | RFS | 267 months |
| Yang, et al., 2008 | p53, Ki-67 | 1986 - 2004 | Korea | 74 | 17 - 77 | 47 | GI, GII, GIII | IHC | OS, RFS | 154.5 months |
| **Study** | **Biomarkers** | **Year of recruitment** | **Country** | **Sample size** | **Age range (years)** | **Female (%)** | **WHO grade** | **Method** | **Outcomes** | **Maximum follow-up** |
| Yoon, et al., 2015 | Ki-67 | 2000 - 2010 | USA | 158 | 19 - 90 | 54 | GII | IHC | PFS | 157 months |
| Zhang, et al., 2022 | CD8^+^TIL, PD-L1 | 2011 – 2021 | China | 93 | 21 – 79 | 72 | GI, GII, GIII | IHC | RFS | 112 months |
| Zhu, et al., 2015 | Ki-67 | 2003 - 2008 | China | 63 | 20 - 79 | 44 | GI, GII, GIII | IHC | RFS | 124 months |

GI, WHO grade I; GII, WHO grade II; GIII, WHO grade III; TMA IHC, Tissue microarray immunohistochemistry; NA, Not available
