# Supplementary material for: Biomarkers for prognosis of meningioma patients: A systematic review and meta-analysis
Source: PLoS One. 2024 May 17;19(5):e0303337. doi: 10.1371/journal.pone.0303337 (PMC11101050; doi:10.1371/journal.pone.0303337)
Supplement: S3 Table — (DOCX) [file pone.0303337.s005.docx]

**S3 Table. Quality assessments of included studies using QUIPS six domains**

| **Study** | **Study participation** | **Study attrition** | **Prognostic Factor Measurement** | **Outcome Measurement** | **Study Confounding** | **Statistical Analysis and Reporting** | **Overall Risks of Bias** |
| --- | --- | --- | --- | --- | --- | --- | --- |
| Abdelazaher, et al., 2011 | Moderate | Moderate | Low | Moderate | Low | Low | Moderate |
| Abdelzaher, et al., 2013 | Low | Moderate | Low | Moderate | Low | Low | Moderate |
| Ammendola, et al., 2022 | Moderate | Moderate | Low | Low | Low | Low | Moderate |
| Anand, et al., 2022 | Low | Moderate | Low | Low | Low | Low | Low |
| Assimakopoulou, et al., 2023 | Low | Moderate | Low | Moderate | High | Low | High |
| Barresi, et al., 2006 | Low | Moderate | Low | Moderate | Low | Low | Moderate |
| Barresi, et al., 2015 | Moderate | Moderate | Low | Low | Low | Low | Moderate |
| Baumgarten, et al., 2016 | Moderate | Moderate | Low | Moderate | High | Low | High |
| Behling, et al., 2021 | Low | Moderate | Low | Low | Low | Low | Low |
| Behling, et al., 2023 | Low | Moderate | Low | Moderate | Low | Low | Moderate |
| Bruna, et al., 2007 | Low | Moderate | Low | Low | Low | Low | Low |
| Cai, et al., 2017 | Low | Moderate | Low | Low | Low | Low | Low |
| Cardona, et al., 2019 | Low | Moderate | Low | Moderate | High | Low | High |
| Champeaux, et al., 2016 | Low | Low | Low | Low | Low | Low | Low |
| Champeaux, et al., 2017 | Low | Low | Low | Low | Low | Low | Low |
| Chang, et al., 2023 | Low | Moderate | Low | Moderate | Low | Low | Moderate |
| Chen, et al., 2021 | Low | Moderate | Low | Low | Low | Low | Low |
| Choi, et al., 2018 | Low | Moderate | Low | Low | Low | Low | Low |
| Damen, et al., 2021 | Low | Moderate | Low | Moderate | Low | Low | Moderate |
| Deguchi, et al., 2020 | Low | Low | Low | Moderate | Low | Low | Low |
| Di Bonaventura, et al., 2022 | Low | Moderate | Low | Low | High | Low | High |
| Endo, et al., 2016 | Low | Low | Low | Low | Low | Low | Low |
| Gauchotteet, et al., 2017 | Low | Moderate | Moderate | Moderate | Low | Low | Moderate |
| Gauchotte, et al., 2020 | Low | Moderate | Low | Low | Low | Low | Low |
| Gauchotteet, et al., 2023 | Low | High | Low | Moderate | Moderate | Low | High |
| **Study** | **Study participation** | **Study attrition** | **Prognostic Factor Measurement** | **Outcome Measurement** | **Study Confounding** | **Statistical Analysis and Reporting** | **Overall Risks of Bias** |
| Gousias, et al., 2014 | Low | Moderate | Low | Low | Low | Low | Low |
| Guadagno, et al., 2016 | Low | Moderate | Low | Low | Low | Low | Low |
| Guillaudeau, et al., 2012 | Low | Moderate | Low | Moderate | Low | Low | Moderate |
| Han, et al., 2016 | Low | Moderate | Low | Low | Low | Low | Low |
| Hsu, et al., 1998 | Low | Moderate | Low | Low | Low | Low | Low |
| Hua, et al., 2017 | Low | Moderate | Low | Moderate | Low | Low | Moderate |
| Hua, et al., 2020 | Low | Low | Low | Moderate | Low | Low | Low |
| Hua, et al., 2023 | Low | Moderate | Moderate | Low | Low | Low | Moderate |
| Jensen, et al., 2012 | Moderate | Moderate | Low | Low | Low | Low | Moderate |
| Jiang, et al., 2012 | Low | Moderate | Low | Low | Low | Low | Low |
| Jung, et al., 2021 | Low | Moderate | Low | Low | Low | Low | Low |
| Kalala, et al., 2004 | Moderate | Low | Moderate | Low | Low | Low | Moderate |
| Karsy, et al., 2018 | Low | Moderate | Low | Low | Low | Low | Low |
| Katz, et al., 2018 | Low | Low | Low | Low | Low | Low | Low |
| Ke, et al., 2014 | Low | Moderate | Low | Low | Low | Low | Low |
| Kim, et al., 2006 | Low | Moderate | Low | Low | Low | Low | Low |
| Kim, et al., 2007 | Low | Moderate | Low | Low | Low | Low | Low |
| Kim, et al., 2012 | Low | Moderate | Low | Low | Low | Low | Low |
| Kim, et al., 2014 | Low | Moderate | Low | Low | Low | Low | Low |
| Kim, et al., 2018 | Low | Moderate | Low | Low | Low | Low | Low |
| Klinger, et al., 2015 | Low | Moderate | Low | Low | Low | Low | Low |
| Konstantinidou, et al., 2003 | Moderate | Low | Low | Moderate | Low | Low | Moderate |
| Korshunov, et al., 2002 | Low | Moderate | Low | Low | Low | Low | Low |
| Korshunov, et al., 2003 | Low | Moderate | Low | Low | High | Low | High |
| Koschny, et al., 2015 | Moderate | Moderate | Low | Low | High | Low | High |
| Kuo, et al., 2019 | Moderate | Moderate | Low | Moderate | Low | Low | Moderate |
| **Study** | **Study participation** | **Study attrition** | **Prognostic Factor Measurement** | **Outcome Measurement** | **Study Confounding** | **Statistical Analysis and Reporting** | **Overall Risks of Bias** |
| Li, et al., 2016 | Low | Moderate | Moderate | Low | High | Low | High |
| Ling, et al., 2016 | Low | Moderate | Low | Low | Low | Low | Low |
| Liu, et al., 2017 | Low | Moderate | Low | Moderate | Low | Low | Moderate |
| Liu, et al., 2021 | Low | Low | Low | Moderate | High | Low | High |
| Maier, et al., 2020 | Low | Low | Low | Low | High | Low | High |
| Marciscano, et al., 2016 | Low | Moderate | Low | Low | Low | Low | Low |
| Matsuno, et al., 1996 | Moderate | Moderate | Low | Moderate | High | Low | High |
| Mirian, et al., 2020 | Low | Low | Low | Moderate | Low | Low | Low |
| Moutafidi, et al., 2021 | Moderate | Moderate | Low | Low | High | Low | High |
| Nakabayashi, et al., 2003 | Low | Moderate | Low | Moderate | Low | Low | Moderate |
| Nakasu, et al., 2009 | Low | Moderate | Low | Moderate | Low | Low | Moderate |
| Nakya, et al., 2009 | Moderate | Moderate | Low | Low | Low | Moderate | Moderate |
| Nanda, et al., 2016 | Low | Moderate | Low | Moderate | Low | Low | Moderate |
| Nassiri, et al., 2021 | Low | Moderate | Low | Low | Low | Low | Low |
| Nowak-Cho, et al., 2021 | Low | Moderate | Low | Low | Low | Low | Low |
| Ohba, et al., 2011 | Low | Moderate | Low | Low | Low | Low | Low |
| Olar, et al., 2015 | Low | Low | Low | Low | Low | Low | Low |
| Olar, et al., 2017 | Moderate | Moderate | Low | Low | Low | Low | Moderate |
| Oya, et al., 2012 | Low | Moderate | Moderate | Low | Low | Low | Moderate |
| Parada, et al., 2018 | Low | Low | Moderate | Low | High | Low | High |
| Parada, et al., 2020 | Moderate | Moderate | Low | Low | High | Low | High |
| Park, et al., 2022 | Low | Low | Low | Low | Low | Low | Low |
| Perry, et al., 1998 | Moderate | Moderate | Low | Moderate | High | Low | High |
| Potti, et al., 2004 | Moderate | Moderate | Low | Low | High | Low | High |
| Prat-Acin, et al., 2021 | Moderate | Moderate | Low | Moderate | High | Low | High |
| Ren, et al., 2022 | Low | Low | Low | Low | Low | Low | Low |
| Roser, et al., 2004 | Low | Moderate | Low | Low | High | Low | High |
| **Study** | **Study participation** | **Study attrition** | **Prognostic Factor Measurement** | **Outcome Measurement** | **Study Confounding** | **Statistical Analysis and Reporting** | **Overall Risks of Bias** |
| Samal, et al., 2020 | Low | Moderate | Low | Low | Low | Low | Low |
| Sanz, et al., 2013 | Low | Moderate | Low | Low | High | Low | High |
| Shan, et al., 2017 | Low | Low | Low | Low | Low | Low | Low |
| Sun, et al., 2020 | Low | Moderate | Moderate | Moderate | Low | Low | Moderate |
| Toland, et al., 2020 | Low | Low | Low | Moderate | Low | Low | Low |
| Tsai, et al., 2016 | Moderate | Moderate | Moderate | Moderate | High | Low | High |
| Tsai, et al., 2018 | Moderate | Moderate | Low | Moderate | High | Low | High |
| Ulgen, et al., 2019 | Low | Moderate | Low | Moderate | High | Low | High |
| Vaubel, et al., 2023 | Low | Moderate | Low | Moderate | High | Low | High |
| Vranic, et al., 2010 | Low | Moderate | Low | Low | Low | Low | Low |
| Wang, et al., 2012 | Low | Low | Low | Moderate | Low | Low | Low |
| Wang, et al., 2013 | Low | Moderate | Low | Low | Low | Low | Low |
| Wang, et al., 2014 | Low | Moderate | Low | Low | Low | Low | Low |
| Winther, et al., 2016 | Low | Moderate | Low | Low | Low | Low | Low |
| Winther, et al., 2017 | Low | Moderate | Low | Low | Low | Low | Low |
| Yamaguchi, et al., 2014 | Low | Moderate | Low | Low | Low | Low | Low |
| Yamamoto, et al., 2021 | Moderate | Low | Low | Low | High | Low | High |
| Yamasaki, et al., 2000 | Low | Moderate | Low | Low | Low | Low | Low |
| Yang, et al., 2007 | Low | Low | Low | Low | Low | Low | Low |
| Yoon, et al., 2015 | Low | Moderate | Low | Low | Low | Low | Low |
| Zhang, et al., 2022 | Low | Moderate | Low | Low | Low | Low | Low |
| Zhu, et al., 2015 | Low | Moderate | Low | Moderate | Low | Low | Moderate |
