# Supplementary material for: Biomarkers for prognosis of meningioma patients: A systematic review and meta-analysis
Source: PLoS One. 2024 May 17;19(5):e0303337. doi: 10.1371/journal.pone.0303337 (PMC11101050; doi:10.1371/journal.pone.0303337)
Supplement: S4 Table — (DOCX) [file pone.0303337.s006.docx]

**S4 Table. Subgroup analysis of cyclin A on recurrence-free survival of meningioma patients**

| **Biomarkers** | **Outcomes** | **Subgroups** | **No. of studies** | **Statistical model** | **Heterogeneity** | | **Pooled Data** | |
| --- | --- | --- | --- | --- | --- | --- | --- | --- |
|  |  |  |  |  | **P of Cochrane Q statistic** | **I^2^ (%)** | **HR (95% CI)** | **P value** |
| Cyclin A | OS | All | 3 | R | 0.02 | 74 | 4.91 (1.38, 17.44) | 0.01 |
|  |  | WHO grade |  |  |  |  |  |  |
|  |  | Low and high grade | 3 | R | 0.02 | 74 | 4.91 (1.38, 17.44) | 0.01 |
|  |  | Cut-off |  |  |  |  |  |  |
|  |  | ≤3 | 1 | - | - | - | 135.88 (8.3, 2224.08) | 0.0006 |
|  |  | >3 | 1 | - | - | - | 2.4 (1.23, 4.67) | 0.01 |
|  |  | NA | 1 | - | - | - | 3.25 (1.32, 8.02) | 0.01 |

R, random-effects model; HR, hazard ratio; CI, confidence intervals, Low and high grade, Grade I, II and III, or Grade I and II
