# Supplementary material for: Biomarkers for prognosis of meningioma patients: A systematic review and meta-analysis
Source: PLoS One. 2024 May 17;19(5):e0303337. doi: 10.1371/journal.pone.0303337 (PMC11101050; doi:10.1371/journal.pone.0303337)
Supplement: S5 Table — (DOCX) [file pone.0303337.s007.docx]

**S5 Table. Subgroup analysis of H3K27me3 on overall survival, recurrence-free survival, and progression-free survival of meningioma patients**

| **Biomarkers** | **Outcomes** | **Subgroups** | **No. of studies** | **Statistical model** | **Heterogeneity** | | **Pooled Data** |  |
| --- | --- | --- | --- | --- | --- | --- | --- | --- |
|  |  |  |  |  | **P of Cochrane Q statistic** | **I^2^ (%)** | **HR (95% CI)** | **P value** |
| H3K27me3 | OS | All | 4 | R | <0.00001 | 90 | 1.05 (0.40, 2.78) | 0.92 |
|  |  | WHO grade |  |  |  |  |  |  |
|  |  | Low and high grade | 2 | R | 0.0006 | 92 | 0.45 (0.14 – 1.48) | 0.19 |
|  |  | High grade | 2 | R | 0.78 | 0 | 2.81 (1.50, 5.26) | 0.001 |
|  | RFS | All | 5 | R | 0.003 | 75 | 1.86 (0.88, 3.91) | 0.1 |
|  |  | WHO grade |  |  |  |  |  |  |
|  |  | Low and high grade | 3 | R | 0.005 | 81 | 1.34 (0.61, 2.94) | 0.47 |
|  |  | High grade | 2 | R | 0.24 | 28 | 5.42 (1.25, 23.45) | 0.02 |
|  | PFS | All | 4 | R | 0.006 | 76 | 0.98 (0.43, 2.22) | 0.97 |
|  |  | WHO grade |  |  |  |  |  |  |
|  |  | Low and high grade | 4 | R | 0.006 | 76 | 0.98 (0.43, 2.22) | 0.97 |

R, random-effects model; HR, hazard ratio; CI, confidence intervals, Low and high grade, Grade I, II and III, or Grade I and II, High grade, Grade II and III, or Grade II or Grade III
