# Supplementary material for: Biomarkers for prognosis of meningioma patients: A systematic review and meta-analysis
Source: PLoS One. 2024 May 17;19(5):e0303337. doi: 10.1371/journal.pone.0303337 (PMC11101050; doi:10.1371/journal.pone.0303337)
Supplement: S6 Table — (DOCX) [file pone.0303337.s008.docx]

**S6 Table. Subgroup analysis of PHH3 on recurrence-free survival of meningioma patients**

| **Biomarkers** | **Outcomes** | **Subgroups** | **No. of studies** | **Statistical model** | **Heterogeneity** | | **Pooled Data** | |
| --- | --- | --- | --- | --- | --- | --- | --- | --- |
|  |  |  |  |  | **P of Cochrane Q statistic** | **I^2^ (%)** | **HR (95% CI)** | **P value** |
| PHH3 | RFS | All | 5 | R | <0.00001 | 94 | 1.11 (1.0, 1.24) | 0.05 |
|  |  | WHO grade |  |  |  |  |  |  |
|  |  | Low and high grade | 4 | R | <0.00001 | 95 | 1.11 (0.99, 1.26) | 0.08 |
|  |  | High grade | 1 | - | - | - | 1.12 (0.94, 1.33) | 0.21 |
|  |  | Cut-off |  |  |  |  |  |  |
|  |  | Category | 3 | R | <0.00001 | 94 | 2.75 (0.77, 9.76) | 0.12 |
|  |  | Continuous value | 2 | R | 0.14 | 53 | 1.02 (0.91, 1.14) | 0.78 |

R, random-effects model; HR, hazard ratio; CI, confidence intervals, Low and high grade, Grade I, II and III or Grade I and II, High grade, Grade II and III, or Grade II or Grade III
