# Supplementary material for: Biomarkers for prognosis of meningioma patients: A systematic review and meta-analysis
Source: PLoS One. 2024 May 17;19(5):e0303337. doi: 10.1371/journal.pone.0303337 (PMC11101050; doi:10.1371/journal.pone.0303337)
Supplement: S7 Table — (DOCX) [file pone.0303337.s009.docx]

**S7 Table. Subgroup analysis of Ki-67 on overall survival, recurrence-free survival, and progression-free survival of meningioma patients**

| **Biomarkers** | **Outcomes** | **Subgroups** | **No. of studies** | **Statistical model** | **Heterogeneity** | | **Pooled Data** | |
| --- | --- | --- | --- | --- | --- | --- | --- | --- |
|  |  |  |  |  | **P of Cochrane Q statistic** | **I^2^ (%)** | **HR (95% CI)** | **P value** |
| Ki-67/MIB-1 | OS | All | 16 | R | <0.00001 | 82 | 1.03 (1.02, 1.05) | <0.0001 |
|  |  | WHO grade |  |  |  |  |  |  |
|  |  | Low and high grade | 8 | R | <0.00001 | 84 | 1.01 (1.0, 1.03) | 0.03 |
|  |  | Low grade | 1 | - | - | - | 0.94 (0.72, 1.23) | 0.65 |
|  |  | High grade | 7 | R | 0.06 | 50 | 1.11 (1.03, 1.19) | 0.005 |
|  |  | Cut-off |  |  |  |  |  |  |
|  |  | ≤ 4 | 5 | R | 0.16 | 40 | 1.15 (0.93, 1.42) | 0.2 |
|  |  | >4 | 8 | R | <0.0001 | 80 | 1.96 (1.29, 2.96) | 0.002 |
|  |  | Continuous value | 3 | R | 0.0001 | 89 | 1.01 (1.0, 1.02) | 0.007 |
|  | RFS | All | 35 | R | <0.00001 | 84 | 1.33 (1.21, 1.46) | <0.00001 |
|  |  | WHO grade |  |  |  |  |  |  |
|  |  | Low and high grade | 15 | R | <0.00001 | 82 | 1.24 (1.10, 1.39) | 0.003 |
|  |  | Low grade | 5 | R | 0.33 | 13 | 3.38 (2.13, 5.36) | <0.00001 |
|  |  | High grade | 15 | R | <0.00001 | 86 | 1.40 (1.12, 1.74) | 0.003 |
|  |  | Cut-off |  |  |  |  |  |  |
|  |  | ≤ 4 | 3 | R | 0.35 | 4 | 4.1 (2.14, 7.83) | <0.0001 |
|  |  | >4 | 22 | R | <0.00001 | 85 | 1.55 (1.30, 1.85) | <0.00001 |
|  |  | Continuous value | 10 | R | <0.0001 | 76 | 1.08 (0.98, 1.2) | 0.12 |
|  | PFS | All | 13 | R | <0.00001 | 83 | 1.02 (1.0, 1.04) | 0.01 |
|  |  | WHO grade |  |  |  |  |  |  |
|  |  | Low and high grade | 6 | R | <0.00001 | 87 | 1.01 (0.99, 1.02) | 0.32 |
|  |  | Low grade | 2 | R | 0.04 | 76 | 1.26 (0.87, 1.84) | 0.23 |
|  |  | High grade | 5 | R | <0.0001 | 85 | 1.85 (1.14, 3.0) | 0.01 |
|  |  | Cut-off |  |  |  |  |  |  |
|  |  | ≤ 4 | 4 | R | 0.0001 | 85 | 1.91 (1.1, 3.29) | 0.02 |
|  |  | >4 | 7 | R | <0.00001 | 86 | 1.98 (1.3, 3.02) | 0.002 |
|  |  | Continuous value | 2 | R | 0.24 | 28 | 1.0 (1.0, 1.01) | <0.0001 |

R, random-effects model; HR, hazard ratio; CI, confidence intervals, Low and high grade, Grade I, II and III or Grade I and II, Low grade, Grade I, High grade, Grade II and III or Grade II or Grade III
